# Coordination of Cell Proliferation and Cell Fate Determination by CES-1 Snail

**DOI:** 10.1371/journal.pgen.1003884

**Published:** 2013-10-31

**Authors:** Bo Yan, Nadin Memar, Julia Gallinger, Barbara Conradt

**Affiliations:** 1 Center for Integrated Protein Science, Department of Biology II, Ludwig-Maximilians-University, Munich, Planegg-Martinsried, Germany; 2 Department of Genetics, MCB Graduate Program, Geisel School of Medicine at Dartmouth, Hanover, New Hampshire, United States of America; Stanford University Medical Center, United States of America

## Abstract

The coordination of cell proliferation and cell fate determination is critical during development but the mechanisms through which this is accomplished are unclear. We present evidence that the Snail-related transcription factor CES-1 of *Caenorhabditis elegans* coordinates these processes in a specific cell lineage. CES-1 can cause loss of cell polarity in the NSM neuroblast. By repressing the transcription of the BH3-only gene *egl-1*, CES-1 can also suppress apoptosis in the daughters of the NSM neuroblasts. We now demonstrate that CES-1 also affects cell cycle progression in this lineage. Specifically, we found that CES-1 can repress the transcription of the *cdc-25.2* gene, which encodes a Cdc25-like phosphatase, thereby enhancing the block in NSM neuroblast division caused by the partial loss of *cya-1*, which encodes Cyclin A. Our results indicate that CDC-25.2 and CYA-1 control specific cell divisions and that the over-expression of the *ces-1* gene leads to incorrect regulation of this functional ‘module’. Finally, we provide evidence that *dnj-11* MIDA1 not only regulate CES-1 activity in the context of cell polarity and apoptosis but also in the context of cell cycle progression. In mammals, the over-expression of Snail-related genes has been implicated in tumorigenesis. Our findings support the notion that the oncogenic potential of Snail-related transcription factors lies in their capability to, simultaneously, affect cell cycle progression, cell polarity and apoptosis and, hence, the coordination of cell proliferation and cell fate determination.

## Author Summary

Animal development is a complex process and requires the coordination in space and time of various processes. These processes include the controlled production of cells, also referred to as ‘cell proliferation’, and the adoption by cells of specific fates, also referred to as ‘cell fate determination’. The observation that uncontrolled cell proliferation and cell fate determination contribute to conditions such as cancer, demonstrates that a precise coordination of these processes is not only important for development but for the prevention of disease throughout life. Snail-related transcription factors have previously been shown to be involved in the regulation of cell proliferation and cell fate determination. For example, the *Caenorhabditis elegans* Snail-related protein CES-1 affects cell fate determination in a specific cell lineage, the NSM (neurosecretory motorneuron) lineage. We now present evidence that CES-1 also controls cell proliferation in this lineage. Within a short period of time, CES-1 therefore coordinates cell proliferation and cell fate determination in one and the same lineage. Based on this finding, we propose that CES-1 is an important coordinator that is involved in the precise control - in space (NSM lineage) and time (<150 min) - of processes that are critical for animal development.

## Introduction

Members of the Snail superfamily of zinc-finger transcription factors are essential during development and their deregulation has been implicated in various malignancies including tumorigenesis [1]–[4]. One of the best known functions of Snail-related proteins is their role in the induction of epithelial-mesenchymal transitions (EMTs) [1], [2], [4], [5]. EMTs are fundamentally important for normal development and, in particular, for processes such as mesoderm formation, gastrulation and neural tube formation. EMTs are also important for tumorigenesis since they are responsible for the invasive behavior of certain types of tumor cells [1], [2], [5]. Hallmarks of EMTs are the loss of apico-basal polarity and adhesive properties, which is critical for the ability of epithelial cells to become migratory. Snail-related proteins contribute to these cellular changes by repressing the transcription of genes that encode factors required for apico-basal polarity and cell adhesion, such as *Crumbs* and *E-cadherin*, respectively [6]–[8].

Snail-related proteins have additional cellular functions that can operate independently of the induction of EMT. In *Drosophila melanogaster*, for example, the Snail family members Snail, Worniu and Escargot are important for both the cell polarity of neuroblasts and the ability of these cells to divide [9], [10]. Snail, Worniu and Escargot are required for the polarity of embryonic neuroblasts because they promote the expression of the gene *inscuteable*, which encodes an adaptor protein that, by forming a physical link between the proteins Par3 and Pins, is thought to connect cell polarity to spindle position [10]–[12]. In the case of the division of neuroblasts, Snail, Worniu and Escargot are thought to enhance cell cycle progression by promoting the expression of the gene *cdc25^string^*, which encodes a Cdc25 phosphatase homolog required for the removal of inhibitory phosphates on Cyclin-dependent kinases (CDKs) and, hence, CDK activation [10], [13], [14]. However, whether the effect of Snail, Worniu and Escargot on *cdc25^string^* is direct or indirect remains to be determined. In mammals, Snail-related proteins have also been shown to regulate cell proliferation [4]. Specifically, a reduced rate of cell proliferation is observed in cultured epithelial cells transfected with *Snail1* (formerly referred to as ‘*Snail*’) and in regions of the mouse embryo that express endogenous *Snail1* [15]. The inhibitory effect of *Snail1* expression on cell proliferation is due to the ability of the Snail1 protein to directly repress the transcription of the *cyclin D2* gene, which is required for the G1 to S phase transition [15]. In the same study, an inverse correlation was also found between *Snail1* expression and apoptosis in the mouse embryo, suggesting that Snail1 can repress apoptosis. Additional evidence that Snail-related transcription factors can repress apoptosis in mammals comes from studies on radiation-induced apoptosis in hematopoietic precursor cells. Snail2 (formerly referred to as ‘Slug’) was found to block apoptosis by repressing the transcription of the pro-apoptotic BH3-only gene *puma* [16].

The ability of Snail-related transcription factors to block apoptosis was initially discovered in *Caenorhabditis elegans* and during the analysis of the NSM (NSM, neuro-secretory motoneuron) lineages (Two bilaterally symmetric NSM lineages exist, the left and right NSM lineage). About 410 min after the 1^st^ division of the embryo (referred throughout the manuscript as “1^st^ round of division”), the two NSM neuroblasts (which are generated about 280 min after the 1^st^ division) divide asymmetrically along the ventral-lateral dorsal-medial axis to each generate two daughter cells of different sizes and different cell fates, the larger NSM, which survives and differentiates into a serotonergic neuro-secretory motorneuron, and the smaller NSM sister cell, which undergoes apoptosis and forms a cell corpse about 30 min after the completion of the NSM neuroblast division [17], [18]. A dominant gain-of-function (gf) mutation of the *ces-1* (ces, cell-death specification) gene, which encodes a Snail-related protein, was found to block the death of the NSM sister cells and the I2 sister cells [19], [20]. Otherwise, *ces-1(n703*gf*)* animals are indistinguishable from wild-type animals at least at the dissecting microscope level. This *ces-1* gf mutation affects a regulatory region of the *ces-1* locus, which is likely to results in the over-expression of the *ces-1* gene in specific lineages, including the NSM lineage [20]. In *ces-1* gf mutants, the CES-1 protein blocks the death of the NSM sister cells by binding to a *cis*-acting element of the BH3-only gene *egl-1* (egl, egg-laying defective), thereby preventing the HLH-2/HLH-3- (HLH, basic helix-loop-helix transcription factor) dependent activation of *egl-1* transcription [21]. Interestingly, the *ces-1* gf mutation also affects the cell polarity of the NSM neuroblast. Specifically, in *ces-1* gf mutant animals, the NSM neuroblast divides symmetrically along randomly selected axes rather than dividing asymmetrically along the ventral-lateral dorsal-medial axis [17]. The same polarity defect is observed in animals that lack a functional *ces-2* or *dnj-11* (dnj, DnaJ domain) gene, which encode a HLF-like bZIP transcription and a MIDA1-like chaperone, respectively, and which act upstream of *ces-1* to repress *ces-1* transcription in the NSM neuroblast [17], [19], [20], [22]. Furthermore, in a wild-type background, expression from a functional P*_ces-1_ces-1::yfp* construct is detected only in the larger NSM daughter that is destined to survive (the NSM); however, in a *ces-2* or *dnj-11* mutant background expression of this construct is detected in the NSM neuroblast as well as both daughter cells [17]. Based on these findings it has been proposed that in the NSM neuroblast, CES-2 and DNJ-11 maintain *ces-1* expression below a certain level and that this is important for the establishment and/or maintenance of NSM neuroblast polarity and the ability of the NSM neuroblast to divide asymmetrically. After the NSM neuroblast divides, CES-1 protein is restricted to the NSM, where it acts as a direct repressor of *egl-1* transcription and hence, apoptosis. In *ces-2*, *dnj-11* or *ces-1* gf mutant animals, the level of CES-1 protein in the NSM neuroblast is elevated and this leads to the symmetric, random division of the NSM neuroblast. This results in the formation of two daughters of similar sizes, both of which contain CES-1 protein and, consequently, survive [17], [23].

We now demonstrate that CES-1 has an additional function in the NSM lineage. Specifically, we present evidence that CES-1 can also regulate cell cycle progression in the NSM neuroblast by functionally interacting with core components of the cell cycle machinery. By simultaneously controlling cell cycle progression, cell polarity and apoptosis, the Snail-related transcription factor CES-1 plays a crucial role in the coordination of cell proliferation and cell fate specification in the NSM lineage.

## Results

### In *ces-1(n703*gf*); bc416* animals, the division of the NSM neuroblast is blocked

**Figure 1:**
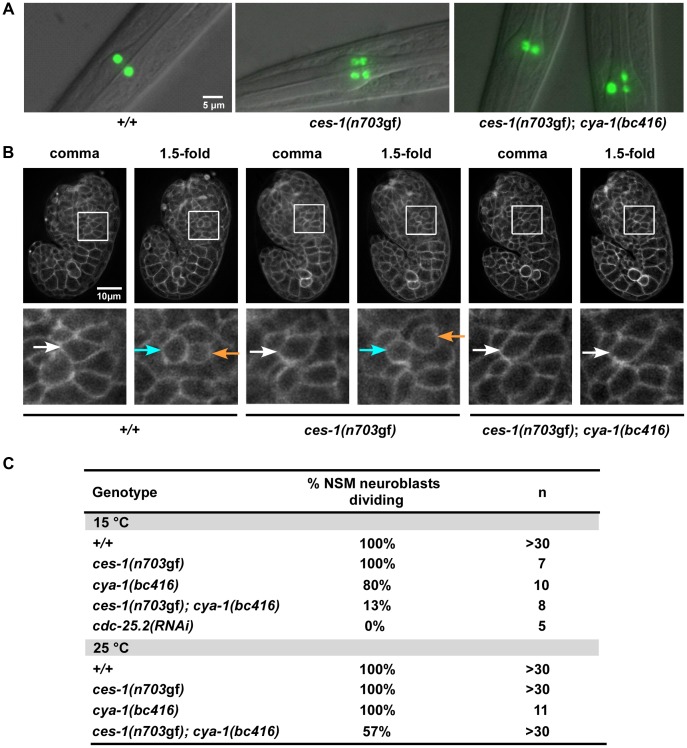
*ces-1(n703*gf*)*; *cya-1(bc416)* affects the division of the NSM neuroblast. (A) The presence of NSMs, undead NSM sister cells, and non-dividing NSM neuroblasts was analyzed in L3, L4 larvae using the reporter P*_tph-1_his-24::gfp* (*bcIs66*). All strains analyzed were homozygous for *bcIs66*. Epifluorescence images overlaid with DIC. (B) The NSM neuroblast division was analyzed in embryos using the reporter P*_pie-1_mCherry::ph^PLC1δ^* (*ltIs44*) or P*_pie-1_gfp::ph^PLC1δ^* (*ltIs38*). Epifluorescence images were taken before (‘comma’) and after the NSM neuroblast division (‘1.5-fold’). In the case of *ces-1(n703*gf*)*; *cya-1(bc416)*, the NSM neuroblasts had not divided at the time the analysis had to be terminated due to the beginning of muscle twitching (around the 2-fold stage). White arrows point to the NSM neuroblasts, blue and orange arrows point to the NSMs and NSM sister cells, respectively. All strains analyzed were homozygous for *ltIs44* and *bcIs66*, except *ces-1(n703*gf*)*, which was homozygous for *ltIs38* and *bcIs66*. (C) Quantification of the percentage of NSM neuroblasts dividing in wild-type, *ces-1(n703*gf*)*, *ces-1(n703*gf*)*; *cya-1(bc416)*, *cya-1(bc416)* and *cdc-25.2(RNAi)* embryos. *cdc-25.2(RNAi)* was performed by injection. n indicates the number of NSM neuroblasts analyzed.

Wild-type larvae carrying the P*_tph-1_his-24::gfp* reporter (tph, tryptophane hydroxylase; his, histone structural gene), which is specifically expressed in serotonergic neurons (and labels the nuclei of these neurons) [24], have two GFP-positive neurons in the anterior pharynx, the left and right NSM (Figure 1A, +/+). In animals carrying a gf mutation of *ces-1*, *n703*, the NSM neuroblast divides symmetrically, resulting in two daughter cells of similar sizes, both of which survive [17], [19]. Therefore, *ces-1(n703*gf*)* larvae carrying the P*_tph-1_his-24::gfp* reporter have four GFP-positive neurons in the head region, the left and right NSM and the left and right ‘undead’ NSM sister cell (Figure 1A, *ces-1(n703*gf*)*). To identify targets of the CES-1 protein involved in the asymmetric division of the NSM neuroblast, we performed a *ces-1(n703*gf*)* suppressor screen using the P*_tph-1_his-24::gfp* reporter as a tool. Specifically, we screened mutagenized *ces-1(n703*gf*)* animals for mutations that cause a reduction in the number of GFP-positive NSMs and undead NSM sister cells (*i.e.* less than four GFP-positive cells in the anterior pharynx). Using this approach, we isolated the mutation *bc416*. At 15°C, 100% of *ces-1(n703*gf*)* larvae have four GFP-positive cells. In contrast, only 5% of *ces-1(n703*gf*)* larvae homozygous for *bc416* (*ces-1(n703*gf*)*; *bc416*) have four GFP-positive cells (Table 1). The *bc416* mutation is recessive and does not show maternal rescue (data not shown; Table S1).

**Table 1:** *ces-1(n703*gf*); cya-1(bc416)* affects the number of ‘NSM-like’ cells.

Genotype	Transgene	Line	% worms with X numbers of GFP^+^ cells			n	% NSM neuroblasts dividing
			2	3	4		
*+/+*	-	-	100	0	0	>200	100%
*ces-1(n703*gf*)*	-	-	0	0	100	>200	100%
*ces-1(n703*gf*); cya-1(bc416)*	-	-	62	33	5	>200	22%
*ced-3(n717)* a	-	-	0	0	100	70	100%
*cya-1(bc416); ced-3(n717)* a	-	-	1	15	84	115	92%
*ces-1(n703*gf*); cya-1(bc416); ced-3(n717)* a	-	-	56	37	7	89	25%
*ces-1(tm1036); cya-1(bc416); ced-3(n717)* a	-	-	1	14	85	>200	92%
*cdc-25.2(ok597)/+; cya-1(bc416); ced-3(n717)* a	-	-	49	36	15	41	33%
*dnj-11(tm2859); cya-1(bc416); ced-3(n717)* a *^, ^* b	-	-	18	43	39	51	61%
*ces-1(n703*gf*); control(RNAi)* c	-	-	0	4	96	46	98%
*ces-1(n703*gf*); cya-1(RNAi)* c	-	-	82	18	0	51	9%
*ces-1(n703*gf*); cya-1(bc416)*	*cya-1* d	1	0	9	91	22	95%
	*cya-1* d	2	4	8	88	24	92%
*ces-1(n703*gf*); cya-1(bc416)*	*cdc-25.2*	1	3	29	68	31	82%

Analysis of P*_tph-1_his-24::gfp* positive cells in the anterior pharynx. All strains analyzed were homozygous for the integration *bcIs66* (P*_tph-1_his-24::gfp*) and were raised and analyzed at 15°C. n indicates the number of L3 or L4 larvae analyzed. ‘% NSM neuroblast dividing’ is defined as the percentage of NSM neuroblasts that divide.

a In the cell-death defective *ced-3(n717)* mutants, the NSM sister cells inappropriately survive.

b Embryonic lethality was observed. The percentage shown in the table is the analysis of larvae survived. In *dnj-11(tm2859); cya-1(bc416); ced-3(n717)* animals that arrest during embyogenesis, only 30% of the NSM neuroblasts divide.

c RNAi was carried out by feeding *ces-1(n703*gf*); rrf-3(pk1426)* animals with bacteria expressing dsRNA. *ZK512.1(RNAi)* was used as *control(RNAi)*.

d Line 1 and Line 2 are two independent transgenic lines generated by injecting the rescue DNA fragment into *ces-1(n703*gf*); cya-1(bc416)* mutants. Transgenic animals were used for analysis.

At 15°C, 33% of *ces-1(n703*gf*)*; *bc416* larvae have three GFP-positive cells (Table 1). Interestingly, based on P*_tph-1_his-24::gfp* labeling, in these animals, one nucleus is larger than the other two nuclei (Figure 1A, *ces-1(n703*gf*)*; *bc416*). This phenomenon is observed at a high frequency. Based on this observation, we hypothesized that instead of suppressing the inappropriate survival of NSM sister cells in *ces-1(n703*gf*)* animals, the *bc416* mutation might affect the division of the NSM neuroblasts. To test this, we directly analyzed the division of the NSM neuroblasts in *ces-1(n703*gf*); bc416* embryos. To that end, we used a transgene that expresses a plasma membrane-targeted mCherry fusion protein as a tool [25]. We identified the NSM neuroblasts based on their positions during the comma stage of embryogenesis and tracked their fates until the 2-fold stage, which is the stage during which in wild-type animals, the NSM neuroblasts complete their division and the NSM sister cells undergo apoptosis [17], [18]. We found that at 15°C, only 13% of the NSM neuroblasts divide in *ces-1(n703*gf*); bc416* embryos (Figure 1B, C). To rule out the possibility that the division of the NSM neuroblasts in *ces-1(n703*gf*); bc416* animals is delayed rather than blocked, we scored *ces-1(n703*gf*)*; *bc416* animals in the background of the *ced-3* loss-of-function mutation *n717*, which causes a general block in apoptosis [26]. If the NSM neuroblasts divided in late embryos or in larvae and the resulting NSM sister cells underwent apoptosis, using P*_tph-1_his-24::gfp* as a tool, we should observed an increased number of animals with four GFP-positive cells in the *ced-3(n717)* background. However, we found that the percentage of animals with two, three, or four GFP-positive cells is not affected by *ced-3(n717)* (Table 1, *ces-1(n703*gf*)*; *bc416*; *ced-3(n717)*). Therefore, the reduction in GFP-positive cells observed in *ces-1(n703*gf*); bc416* larvae is the result of a failure of the NSM neuroblasts to divide. For this reason, we are presenting the data acquired in larvae using P*_tph-1_his-24::gfp* not only in the form of ‘% animals with two, three, or four GFP-positive cells’ but also as ‘% NSM neuroblasts dividing’, which is defined as the percentage of the NSM neuroblasts that divide (Table 1).

### The NSM neuroblast division is blocked between S phase and M phase

To further examine the cell cycle defect in *ces-1(n703*gf*); bc416* animals, we determined the relative DNA content in non-dividing NSM neuroblasts. We labeled DNA in *ces-1(n703*gf*)*; *bc416* animals with the fluorescent dye DAPI and measured fluorescence intensity in non-dividing NSM neuroblasts [27], [28], in NSMs and in undead NSM sister cells. We found that in *ces-1(n703*gf*)*; *bc416* mutants with three GFP-positive cells, the average DNA content of the cells with the larger nuclei (presumably the non-dividing NSM neuroblasts) is 1.8 times greater than the average DNA content of the cells with the smaller nuclei (presumably the NSMs and undead NSM sister cells) (Figure S1). Furthermore, in *ces-1(n703*gf*)*; *bc416* animals with two GFP-positive cells, the average DNA content of both cells is about 2-fold higher than that of control pharyngeal muscle cells (data not shown). Taken together, these observations suggest that in *ces-1(n703*gf*)*; *bc416* animals, the NSM neuroblasts complete DNA replication but fail to undergo mitosis. Hence, in this mutant background, the NSM neuroblasts arrest between S phase and M phase. Interestingly, just like NSMs, the non-dividing, tetraploid NSM neuroblasts express the P*_tph-1_his-24::gfp* reporter during larval stages, which suggests that they differentiate into serotonergic neurons.

### *ces-1(n703*gf*); bc416* causes embryonic lethality and sterility

**Figure 2:**
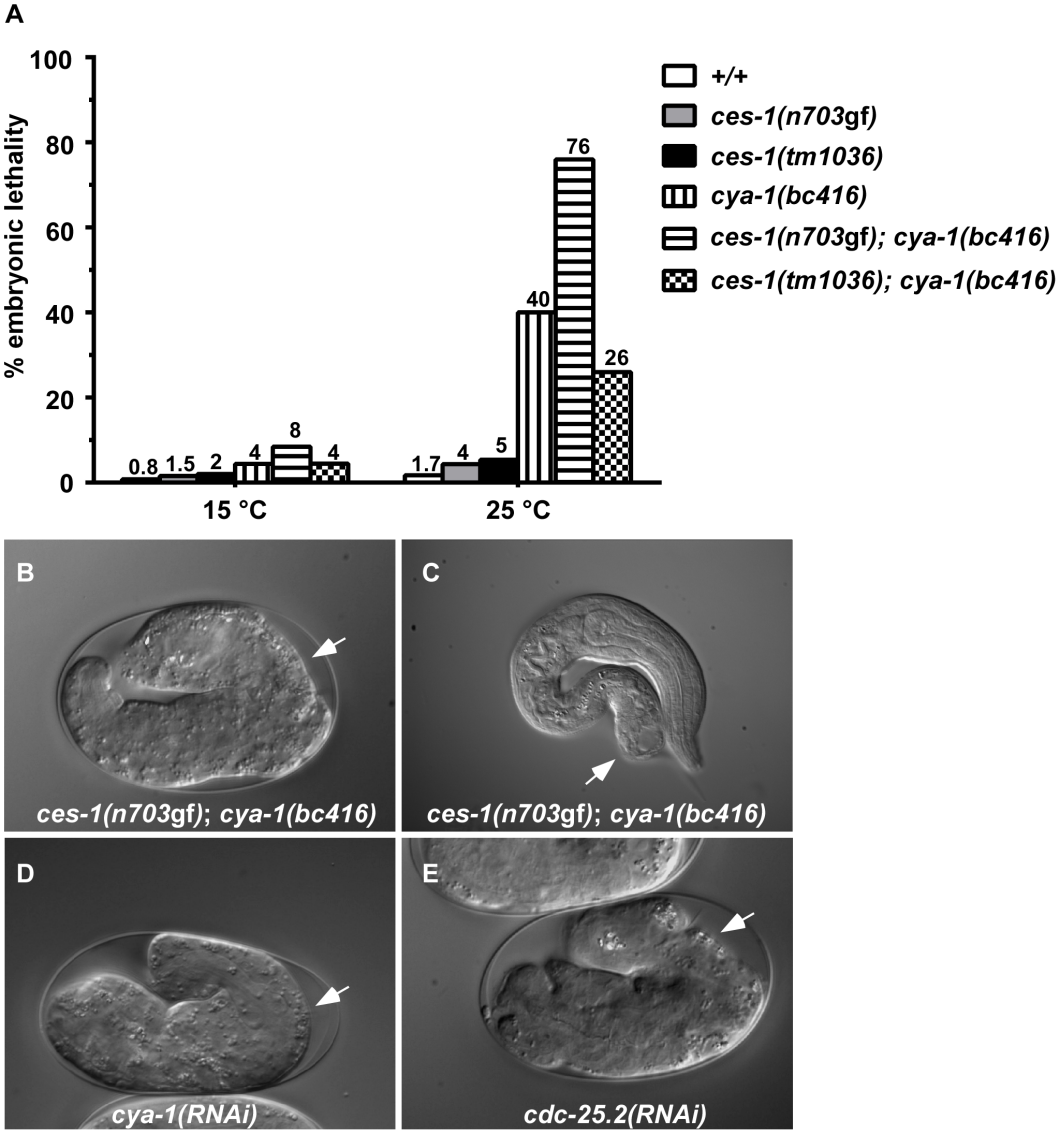
*ces-1(n703*gf*)*; *cya-1(bc416)* causes temperature-sensitive embryonic lethality. (A) The percentages of embryonic lethality at 15°C and 25°C. The numbers above the bars represent the percentage of embryonic lethality. For each genotype, around 1000 embryos were scored. DIC images of embryos arrested during the elongation stage of embryogenesis (B, D, E) or during the first larval stage (L1) (C) when grown at 25°C are shown. White arrows point to abnormalities in the hypodermis. All strains analyzed were homozygous for *bcIs66*. RNAi was performed by injection.

Besides exhibiting a defect in NSM neuroblast division, *ces-1(n703*gf*)*; *bc416* animals have additional defects. When raised at 15°C or 25°C, 8% or 76% of *ces-1(n703*gf*)*; *bc416* animals, respectively, exhibit an embryonic lethal (Emb) phenotype and arrest at the elongation stage during embryogenesis (Figure 2A, B). Arrested embryos have multiple defects in hypodermal morphogenesis (Figure 2B). Since the Emb phenotype is temperature sensitive, we performed temperature-shift experiments to define the temperature-sensitive period (TSP) of *ces-1(n703*gf*)*; *bc416* animals. Embryos were shifted from 25°C to 15°C or *vice versa* at different stages during embryonic development, and viability was assessed 24 h to 48 h later. As shown in Figure S2, the TSP of *ces-1(n703*gf*)*; *bc416* animals lies between the 50-cell stage and the comma stage of embryogenesis. Therefore, at least in the *ces-1(n703*gf*)* mutant background, the gene defined by the *bc416* mutation is essential and its activity required for embryonic development between the 50-cell stage and the comma stage. Furthermore, animals that escape embryonic lethality and hatch also display morphological abnormalities (Figure 2C). Finally, when raised at 25°C, about 30% of *ces-1(n703*gf*)*; *bc416* animals that develop into adults are sterile (Ste phenotype) (data not shown) and the average brood size of the fertile *ces-1(n703*gf*)*; *bc416* adults is smaller than that of wild-type adults (Table S2).

### *ces-1(n703*gf*)*; *bc416* mutants display cell division defects in the ABarp, C, and E lineages

**Figure 3:**
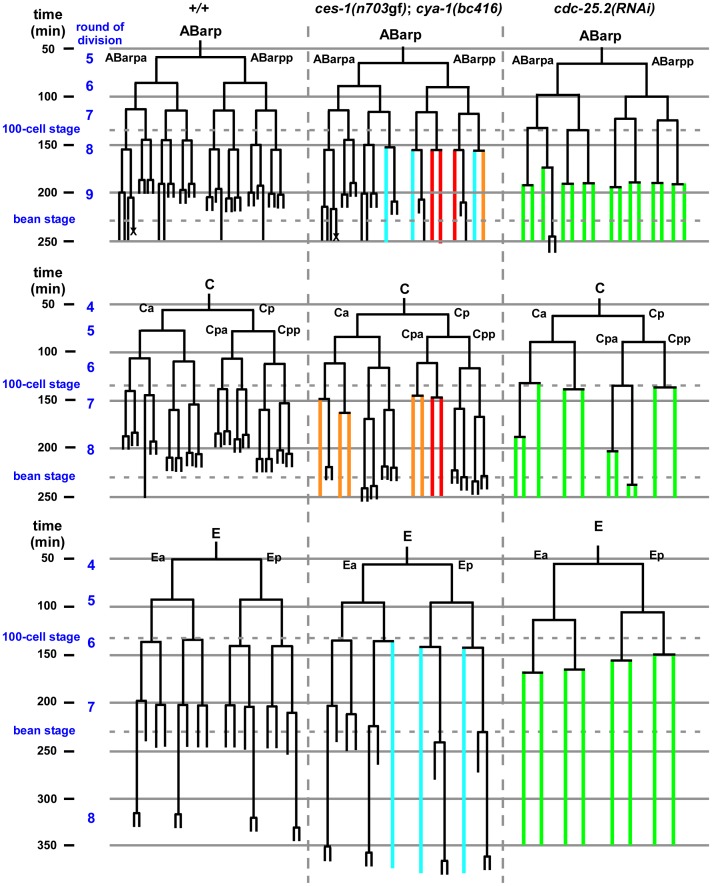
*ces-1(n703*gf*)*; *cya-1(bc416)* blocks cell divisions in the ABarp, C and E lineages. All strains analyzed were homozygous for *bcIs66*. Lineage analyses were performed for two (wild-type, *+/+*), three (*ces-1(n703*gf*); cya-1(bc416)*) and three (*cdc-25.2(RNAi)*) embryos raised at 25°C. The ABarp, C and E lineages are shown. Vertical axis indicates approximate time in min after the 1^st^ round of embryonic division, in which P0 divides into AB and P1. In the case of *ces-1(n703*gf*); cya-1(bc416)*, cell division defects observed in three out of three embryos are depicted in red, defects found in two out of three embryos are depicted in blue, and defects found in one out of three embryos are depicted in orange. In the case of *cdc-25.2(RNAi)*, RNAi was carried out by injection. Since there is some variability of the RNAi effect, the lineage shown here was derived from the embryo with the strongest phenotype (cell division defects observed in this embryo are depicted in green), and the lineages from the other two *cdc-25.2(RNAi)* embryos are shown in Figure S6. The severe cell division defects in the ABarp, C and E lineages were seen in all three *cdc-25.2(RNAi)* embryos. The cell death in the ABarp lineage is labeled with the cross. The defects in the C lineage and ABarp lineage result in a defect in the formation of the hypodermis (the mitoses that generate hyp7, hyp5, hyp11, H0, H1, H2, V1, V2, V4, and V6 fail to occur).

Since a highly penetrant Emb phenotype was observed in *ces-1(n703*gf*); bc416* animals raised at 25°C, we investigated whether cell divisions other than the divisions of the NSM neuroblasts are affected in these animals. A systematic analysis of all cell lineages using 4D lineage analysis [29] showed that cell division defects are not restricted to the NSM lineage. We found that at 25°C, the ABarp, C and E lineages are also affected in *ces-1(n703*gf*)*; *bc416* animals (Figure 3). All other lineages were not affected. ABarp is a major hypodermal precursor and the C founder cell generates additional posterior and dorsal hypodermal cells [18]. In the ABarp lineage, most cell divisions that give rise to ventrolateral ectoblasts (V1 to V6) are blocked (Figure 3). Furthermore, in the C lineage, many cell divisions that generate the embryonic large hypodermal syncytium (hyp7) fail to occur (Figure 3). The defects in the ABarp and C lineage most likely cause or contribute to the observed hypodermal abnormalities (Figure 2B, C). A failure in the formation of the hypodermis has previously been shown to cause embryonic lethality [30]. In addition, the phenotype of arrested *ces-1(n703*gf*); bc416* embryos is similar to the phenotype of mutants with hypodermal defects [30]. Therefore, the cell division defects in the ABarp and C lineages observed in *ces-1(n703*gf*)*; *bc416* animals most probably cause the Emb phenotype exhibited by these animals. In addition, we identified variable defects in the E lineage. Specifically, some cell divisions of the 7^th^ round of division during *C. elegans* embryogenesis do not occur in the E lineage (Figure 3). Based on these observations, we conclude that in the *ces-1(n703*gf*)* background and at 25°C, *bc416* affects the divisions of cells other than the NSM neuroblasts. (The defects caused by *bc416* in an otherwise wild-type background will be discussed below.)

### *bc416* is a mutation in the *C. elegans* Cyclin A homolog, *cya-1*

The *bc416* mutation was mapped genetically to a 900 kb region (between SNPs F22B7:15755 and ZK1098:19075) on LGIII using linkage analysis, three-factor mapping and SNP mapping (Figure S3A). In addition, we used Illumina deep sequencing technology to sequence the entire genome of *ces-1(n703*gf*)*; *bc416* animals. In the F22B7:15755 - ZK1098:19075 region, we found a G to A transition at the conserved 5′ splice-donor site of the first intron of the gene *cya-1* (ZK507.6), which encodes one of two *C. elegans* Cyclin A homologs, CYA-1 (Figure S3B) [31].

A 4.3 kb genomic DNA fragment that contains the entire coding region of *cya-1* rescues the NSM neuroblast division defect (Table 1; *ces-1(n703*gf*)*; *bc416* plus *‘cya-1’* transgene) and the Emb phenotype observed in *ces-1(n703*gf*)*; *bc416* animals (data not shown). Similar to *bc416*, partially reducing *cya-1* function by RNA-mediated interference (RNAi) blocks 90% of the NSM neuroblast divisions in the *ces-1(n703*gf*)* mutant background (Table 1; *ces-1(n703*gf*)*; *cya-1(RNAi)*). In addition, *cya-1*(*RNAi*) leads to embryonic lethality and the terminal phenotype of arrested embryos is similar to the terminal phenotype of arrested *ces-1(n703*gf*); bc416* embryos (Figure 2D). (For the *cya-1* RNAi experiment, sequences of exons 4 and 5 of *cya-1* were used. Sequence alignments reveal that these two exons are highly homologous [≥60%] to *cya-2*, the second *C. elegans* cyclin A gene. For this reason, it is possible that *cya-1(RNAi)* also causes a decrease in *cya-2* function.) Finally, a null mutation of the *cya-1* gene, *he153*, which causes embryonic lethality, fails to complement *bc416* in the *ces-1(n703*gf*)* mutant background (data not shown; S. van der Heuvel, personal communication). In conclusion, the gene defined by *bc416* is identical to the *cya-1* gene.

Since the accuracy of the 5′ splice-donor site is important for the recognition and removal of introns, we determined whether the *bc416* mutation influences the splicing of the primary *cya-1* transcript. Using reverse transcriptase PCR (RT-PCR), we found that, at both 15°C and 25°C, *bc416* affects the splicing of the *cya-1* gene and results in aberrantly spliced messages, in which parts of the first intron are retained (Figure S3D). The translation of these aberrant messages is predicted to result in the synthesis of a truncated, non-functional CYA-1 protein that includes only the first 12 amino acids of the full-length protein. Using quantitative real-time PCR (qPCR), next, we determined the level of correctly spliced, wild-type *cya-1* transcript. We found that at both 15°C and 25°C, compared to wild-type (*cya-1(+/+)*) animals, the level of correctly spliced *cya-1* transcript is reduced by about 50% in *cya-1(bc416)* animals (Figure S3E). These results suggest that *bc416* affects the pre-mRNA splicing of the *cya-1* gene, resulting in a reduction of correctly spliced mRNA and hence, presumably, full-length CYA-1 protein. Therefore, *bc416* most likely represents a partial loss-of-function (lf) mutation of *cya-1*.

### *ces-1(n703*gf*)* enhances the phenotypes caused by *cya-1(bc416)*

While performing *cya-1* RNAi experiments, we noticed that *cya-1* RNAi by injection results in a more penetrant Emb phenotype when performed in the *ces-1(n703*gf*)* background (64% embryonic lethality at 25°C) compared to the wild-type background (40% embryonic lethality at 25°C). To test whether the defects observed in *ces-1(n703*gf*); cya-1(bc416)* animals are dependent on the presence of the *ces-1(n703*gf*)* mutation, we analyzed *bc416* in an otherwise wild-type background. Using the plasma membrane-targeted mCherry fusion protein as a tool, we found that at 15°C, 80% of the NSM neuroblasts divide in *cya-1(bc416)* embryos (Figure 1C). For comparison, only 13% of the NSM neuroblasts divide in *ces-1(n703*gf*); cya-1(bc416)* embryos. Similarly, using the P*_tph-1_his-24::gfp* reporter as a tool, we found that 92% of NSM neuroblasts divide in *cya-1(bc416)* animals in the *ced-3* mutant background (Table 1, *cya-1(bc416)*; *ced-3(n717)*). For comparison, in *ces-1(n703*gf*); cya-1(bc416)* animals in the *ced-3* mutant background only 25% of the NSM neuroblasts divide. Finally, we found that at 25°C, 40% of *cya-1(bc416)* animals arrest at the elongation stage during embryogenesis and therefore exhibit an Emb phenotype (Figure 2A). For comparison, 76% of *ces-1(n703*gf*); cya-1(bc416)* animals exhibited an Emb phenotype when raised at 25°C. Temperature-shift experiments revealed that the TSP of *cya-1(bc416)* animals lies between the 50-cell stage and the comma stage of embryogenesis and therefore is identical to the TSP of *ces-1(n703*gf*); cya-1(bc416)* animals (Figure S2). Lineage analysis revealed that *cya-1(bc416)* embryos have cell division defects in the ABarp, C and E lineages (Figure S4). However, cell division defects in the ABarp and C lineages were only observed in one out of three *cya-1(bc416)* embryos analyzed. For comparison, cell division defects in the ABarp and C lineages were observed in all three *ces-1(n703*gf*); cya-1(bc416)* embryos analyzed. Lineage analysis also revealed that the *ces-1(n703*gf*)* embryos have no cell division defects in the ABarp, C or E lineages (Figure S4). Therefore, *ces-1(n703*gf*)* increases the penetrance of the cell division defects caused by *cya-1(bc416)*, especially in the ABarp and C lineages. This is consistent with the more penetrant Emb phenotype observed in *ces-1(n703*gf*); cya-1(bc416)* animals (76% embryonic lethality at 25°C, Figure 2). These findings demonstrate that *ces-1(n703*gf*)* enhances the NSM neuroblast division defect and the Emb phenotype caused by *cya-1(bc416)*. Interestingly, *ces-1(n703*gf*)* does not enhance the defect in brood size caused by *cya-1*(*bc416*). When raised at 25°C, the brood size of both *cya-1(bc416)* animals and *ces-1(n703*gf*); cya-1(bc416)* animals is reduced by about 2.5-fold when compared to the brood size of wild-type animals (Table S2).

### *ces-1* over-expression reduces the relative expression level of *cdc-25.2*

**Figure 4:**
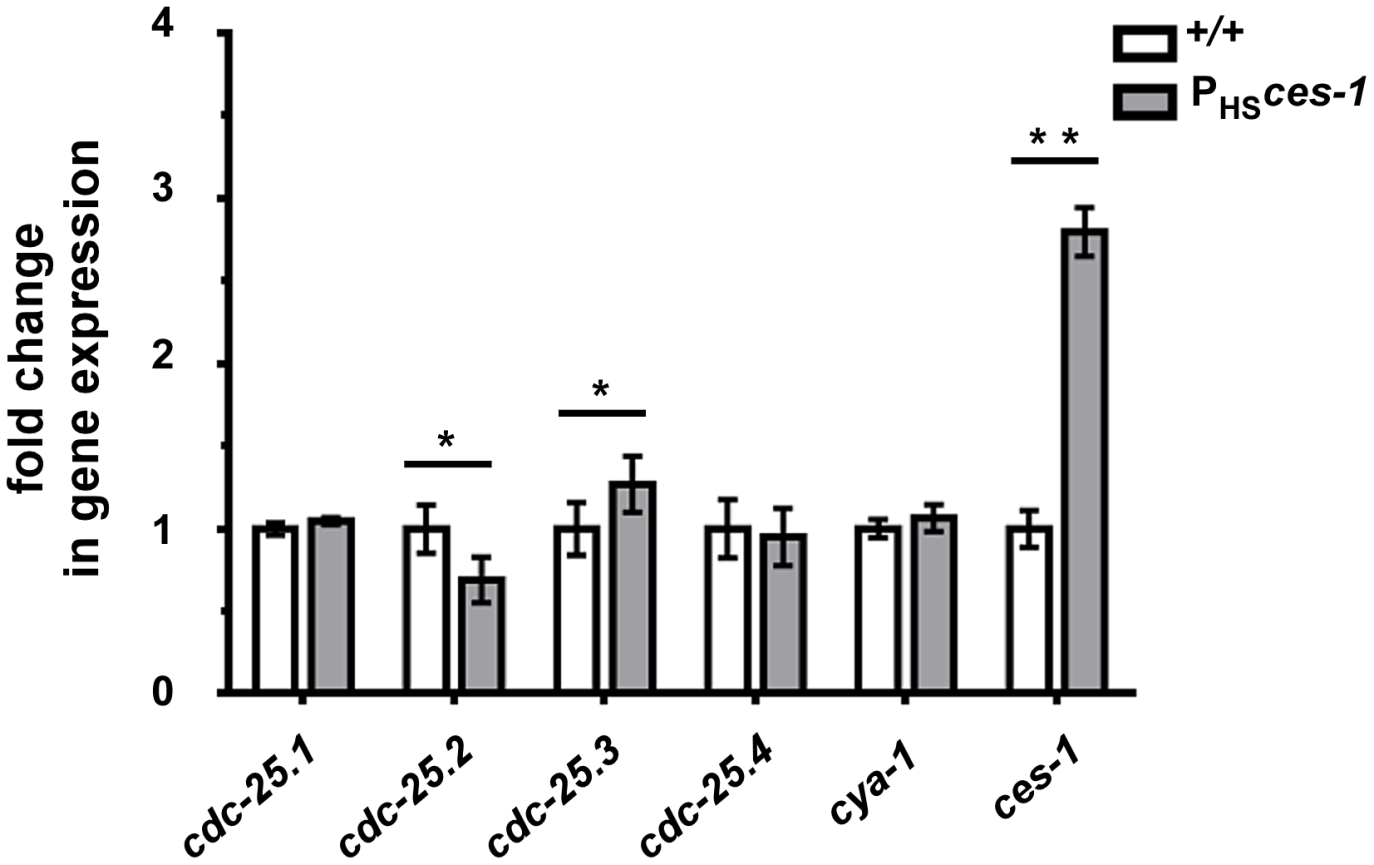
*cdc-25.2* expression is down-regulated by *ces-1* over-expression. Transgenic animals carrying an extra-chromosomal array of *ces-1* heat-shock plasmids and coinjection marker were used as the sample group (P_HS_ *ces-1*), while transgenic animals carrying an extra-chromosomal array of only coinjection marker were used as control (*+/+*). Relative expression levels of *cdc-25* genes and *cya-1* gene in control animals (*+/+*) and animals over-expressing *ces-1* (P_HS_ *ces-1*) were determined by real-time PCR (qPCR). Data are represented as fold change relative to control. Data shown are the means ± SEM from four independent repeats. Paired t-test was used to determine significance. The level of *cdc-25.2* in P_HS_ *ces-1* is significantly lower than in control. The level of *cdc-25.3* in P_HS_ *ces-1* is significantly higher than in control. The levels of *cdc-25.1*, *cdc-25.4*, *cya-1* are not significantly changed in response to *ces-1* over-expression. *p<0.05, **p<0.01 significantly different from the control.

Snail-related transcription factors are thought to predominantly act as repressors of transcription [1]. To determine the mechanism through which *ces-1(n703*gf*)* enhances *cya-1(bc416)*, we therefore analyzed the expression of candidate target genes. The *Drosophila melanogaster* Snail family has been implicated in the control of the expression of the gene *cdc25^string^*, which encodes the *D. melanogaster* ortholog of Cdc25 [10], [13], [14]. For this reason, we analyzed the level of expression of the four *C. elegans cdc25* homologs (*cdc-25.1*, *cdc-25.2*, *cdc-25.3* and *cdc-25.4*) [32] in wild-type animals and in animals over-expressing the *ces-1* gene using qPCR. To that end, using a heat-inducible promoter, *ces-1* expression was induced for 1 h in embryos and embryos were collected after a 1.5 h recovery period. This induction scheme resulted in a 3-fold increase in the relative expression level of *ces-1* (Figure 4). Using this experimental set-up, we found that the relative expression levels of *cdc-25.1* and *cdc-25.4* are not significantly changed in embryos over-expressing *ces-1*. In contrast, the relative expression level of *cdc-25.2* is significantly decreased, indicating that *ces-1* over-expression can repress the expression of *cdc-25.2* (Figure 4). We also found that the relative expression level of *cdc-25.3* is significantly increased. Since CES-1 is thought to predominantly act as repressor of transcription and since it has been suggested that mammalian Cdc25A and Cdc25B may compensate for the loss of Cdc25C [33], [34], we analyzed whether the increase in the relative expression level of *cdc-25.3* in embryos over-expressing *ces-1* is an indirect effect that is triggered by decreased *cdc-25.2* expression. We found that the relative expression level of *cdc-25.3* is not increased in embryos in which *cdc-25.2* function is knocked-down by RNAi (Figure S5). This suggests that, independently of decreasing *cdc-25.2* expression, *ces-1* over-expression increases *cdc-25.3* expression. Finally, the relative expression level of *cya-1* expression is not significantly changed in embryos over-expressing *ces-1* (Figure 4). In summary, our data indicate that CES-1 directly or indirectly represses the expression of *cdc-25.2*.

### CES-1 binds to the *cdc-25.2* locus *in vivo*

**Figure 5:**
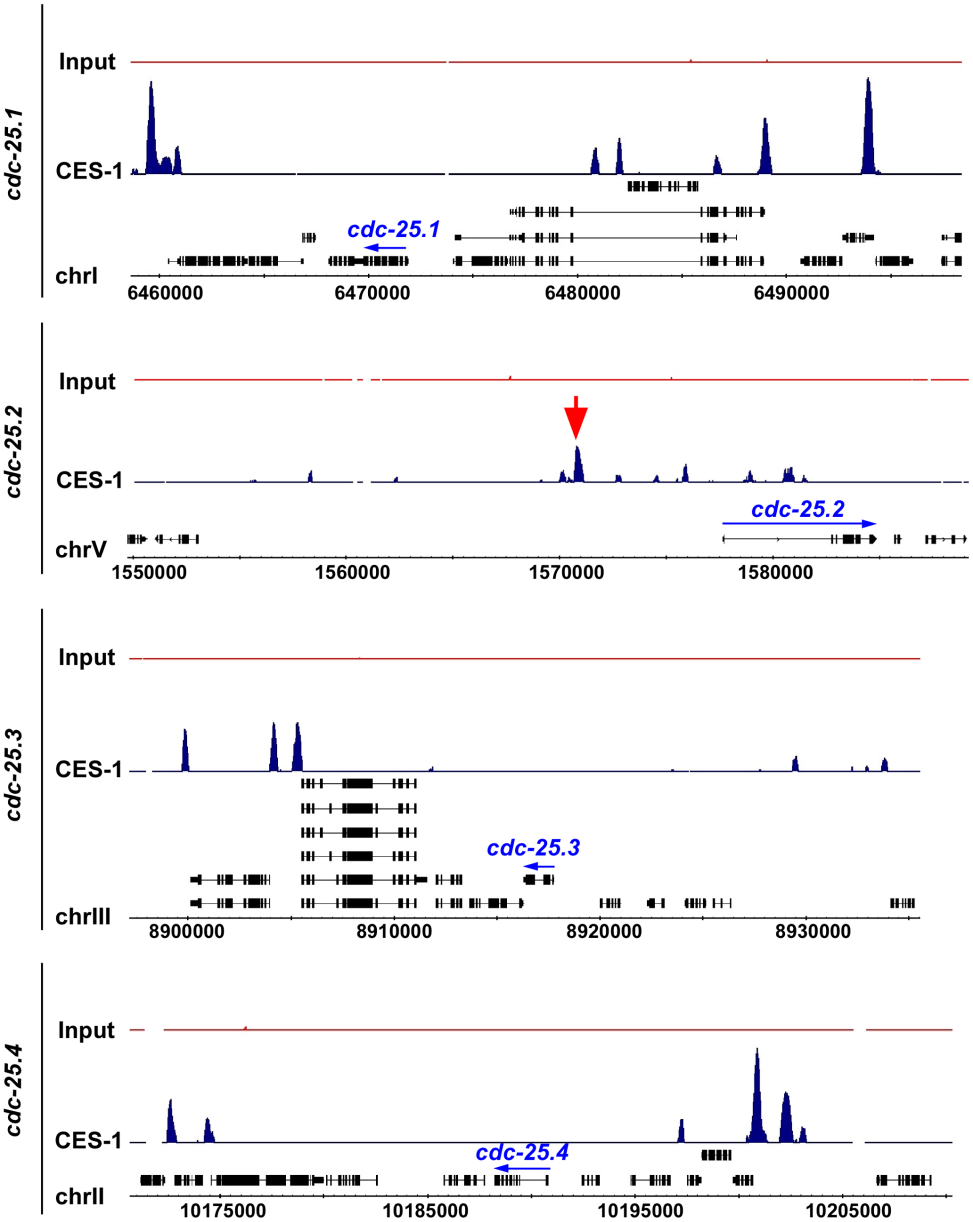
CES-1 binds to an upstream region of the *cdc-25.2* locus. The genome-wide binding sites of the CES-1 protein were identified using ChIP-seq. Shown are the distributions of CES-1-bound regions around the genomic loci of the four *cdc-25* genes, whose transcription units are indicated by blue arrows. The black boxes correspond to the gene exons. The red arrow points to the CES-1-bound region upstream of *cdc-25.2*. Data was visualized using Integrated Genome Browser based on genome WS190 of *C. elegans* [39].

To determine whether CES-1 directly controls the transcription of the *cdc-25.2* gene, we analyzed CES-1 ChIP-seq (ChIP-seq, chromatin immunoprecipitation with massively parallel DNA sequencing) data acquired by the modENCODE Project (http://www.modencode.org/) [35], [36] (M. Snyder, S. Kim, T. Kawli, personal communication). This data was acquired using, as starting material, embryos that harbor multiple copies of an engineered, stably integrated *ces-1* fosmid, which expresses a *ces-1::gfp* transgene under the endogenous *ces-1* promoter [37], [38]. (We have previously shown that a P*_ces-1_ces-1::yfp* transgene can rescue the *ces-1* loss-of-function mutant phenotype and, hence, is generating a fusion protein that is functional [17]. ) The ChIP-seq data obtained indicate that CES-1 binds to a 1.7 kb region that is located 4.8 kb to 6.5 kb upstream of the predicted transcriptional start site of *cdc-25.2* (Figure 5) (Integrated Genome Browser [39]). In contrast, we did not identify peaks indicative of CES-1 binding sites in the immediate regions 5′ or 3′ of the predicted *cdc-25.1*, *cdc-25.3* or *cdc-25.4* transcription units, nor within their introns (Figure 5). Based on these findings we conclude that *cdc-25.2* most likely is a direct target of CES-1 and, hence, that the effect of *ces-1* over-expression on the relative expression level of *cdc-25.2* is a direct effect.

### *cdc-25.2* acts downstream of *ces-1* to promote cell cycle progression

The over-expression of *ces-1* results in a reduction of the relative expression level of *cdc-25.2* by about 30% (Figure 4). To determine whether a decrease in *cdc-25.2* dosage by 50% is sufficient to enhance the NSM neuroblast division defect caused by *cya-1*(*bc416*), we analyzed *cya-1(bc416)* animals heterozygous for *cdc-25.2(ok597)*, a deletion of the *cdc-25.2* gene that removes 2.7 kb of the *cdc-25.2* locus, including four of its six exons [40]. We found that *cdc-25.2(ok597)/+; cya-1(bc416)* animals exhibit a NSM neuroblast division defect similar to the defect observed in *ces-1(n703*gf*); cya-1(bc416)* animals. Specifically, at 15°C, 25% and 33% of the NSM neuroblasts divide in *ces-1(n703*gf*); cya-1(bc416)* or *cdc-25.2(ok597)/+; cya-1(bc416)* animals, respectively (in the *ced-3* mutant background) (Table 1). Conversely, we tested whether the transgenic expression of the *cdc-25.2* transcription unit under the control of the endogenous *cdc-25.2* promoter can rescue the NSM neuroblast division defect observed in *ces-1(n703*gf*); cya-1(bc416)* animals. We found that the expression of *cdc-25.2* significantly reduces the NSM neuroblast division defect in *ces-1(n703*gf*); cya-1(bc416)* animals. Specifically, at 15°C, the expression of *cdc-25.2* increases the percentage of NSM neuroblasts dividing from 22% to 82% (Table 1; *ces-1(n703*gf*)*; *cya-1(bc416)* plus *‘cdc-25.2’* transgene). Together, these findings support the notion that *ces-1(n703*gf*)* enhances the NSM neuroblast division defect caused by *cya-1(bc416)* by decreasing *cdc-25.2* expression.

### *ces-1*, *cdc-25.2* and *cya-1* act together to control cell cycle progression in specific lineages

Next, we analyzed the phenotypes caused by the downregulation of the four *cdc-25* genes. We found that the downregulation by RNAi of *cdc-25.1* or *cdc-25.2* results in embryonic lethality. In contrast, the downregulation by RNAi of *cdc-25.3* or *cdc-25.4* does not cause any obvious abnormalities, which is consistent with previous observations [32]. While *cdc-25.1(RNAi)* embryos arrest during early embryonic stages (as early as the 4-cell stage) (data not shown), *cdc-25.2(RNAi)* embryos arrest at the elongation stage during embryogenesis (Figure 2E, *cdc-25.2(RNAi)*). Using lineage analyses, we determined the phenotype of *cdc-25.2(RNAi)* embryos in more detail. We found that the inactivation of *cdc-25.2* causes an increase in cell cycle length in all lineages (Table S4). For example, in wild-type animals, the average time between the 6^th^ and 7^th^ and between the 7^th^ and 8^th^ round of division in the ABala lineage is 28 min and 36 min, respectively. In *cdc-25.2(RNAi)* animals, the average time is 36 min and 52 min, respectively. In general, many cell divisions of the last three rounds of division during embryonic development are blocked (Figure 3 and Figure S6). For example, the divisions of the NSM neuroblasts are blocked in *cdc-25.2(RNAi)* embryos (Figure 1C). In addition, the ABarp lineage is particularly sensitive to reduced levels of *cdc-25.2* function. Most of the cell divisions during the 9^th^ round of division are blocked in the ABarp lineage (Table S3, Figure 3 and Figure S6). For comparison, in the case of other AB descendants, only half or less than half of the cell divisions during the 9^th^ round of division are blocked (Table S3). We also observed severe cell division defects in the C and E lineages in *cdc-25.2(RNAi)* animals (Figure 3 and Figure S6). Interestingly, the cell lineages that exhibit increased sensitivity to reduced levels of *cdc-25.2* activity are identical to the cell lineages that are affected in *ces-1(n703*gf*); cya-1(bc416)* animals. In summary, these findings support the notion that, in a specific set of lineages, *ces-1, cdc-25.2* and *cya-1* act together to control cell cycle progression. These lineages and cells include the ABarp, C and E lineages as well as the NSM neuroblasts.

Finally, to determine whether the loss of *ces-1* function affects the phenotype caused by *cya-1(bc416)*, we analyzed the NSM neuroblast division in animals homozygous for *cya-1(bc416)* and the *ces-1* deletion allele *tm1036*. *tm1036* is a 1.3 kb deletion that removes exons 2, 3 and 4 of the *ces-1* transcription unit and that is predicted to result in the synthesis of a truncated protein lacking two of the five zinc-finger domains of the CES-1 protein [20]. *ces-1(tm1036)* animals are indistinguishable from wild-type animals at the dissection microscope level, and in an otherwise wild-type background, the loss of *ces-1* function causes no obvious phenotype in the NSM lineage [17], [19], [20]. We found that, in the *ced-3* mutant background, *ces-1(tm1036)* does not suppress the NSM neuroblast division defect caused by *cya-1(bc416)* (Table 1). However, *ces-1(tm1036)* reduces the embryonic lethality caused by *cya-1(bc416)*. While 40% of *cya-1(bc416)* animals exhibit an Emb phenotype, only 26% of *ces-1(tm1036); cya-1(bc416)* animals exhibit an Emb phenotype (Figure 2A). Based on these observations, we suggest that *ces-1* may play a role in the control of cell cycle progression at least in certain cell lineages.

### Genetic interactions between *cya-1* and *dnj-11*

Like *ces-1(n703*gf*)*, the loss of *dnj-11* function causes symmetric NSM neuroblast division and inappropriate NSM sister cell survival [17], [19]. Therefore, we determined whether the loss of *dnj-11* function also enhances the NSM neuroblast division defect caused by *cya-1(bc416)*. We found that animals homozygous for *cya-1(bc416)* and *dnj-11(tm2859)*, a deletion allele of *dnj-11* that removes 614 base pairs of the coding region [17], exhibit a partially penetrant Emb phenotype (data not shown). Whereas in viable larvae 61% of the NSM neuroblasts had divided, only 30% of the NSM neuroblasts divide in *dnj-11(tm2859); cya-1(bc416)* animals that arrest during embyogenesis (at 15°C, Table 1). For comparison, 92% of NSM neuroblasts divide in *cya-1(bc416)* animals raised at 15°C. These findings demonstrate that, like *ces-1(n703*gf*)*, the loss of *dnj-11* function enhances the NSM neuroblast division defect caused by *cya-1(bc416)*. Based on these findings, we conclude that *dnj-11* regulates *ces-1* function also in the context of cell cycle progression.

## Discussion

**Figure 6:**
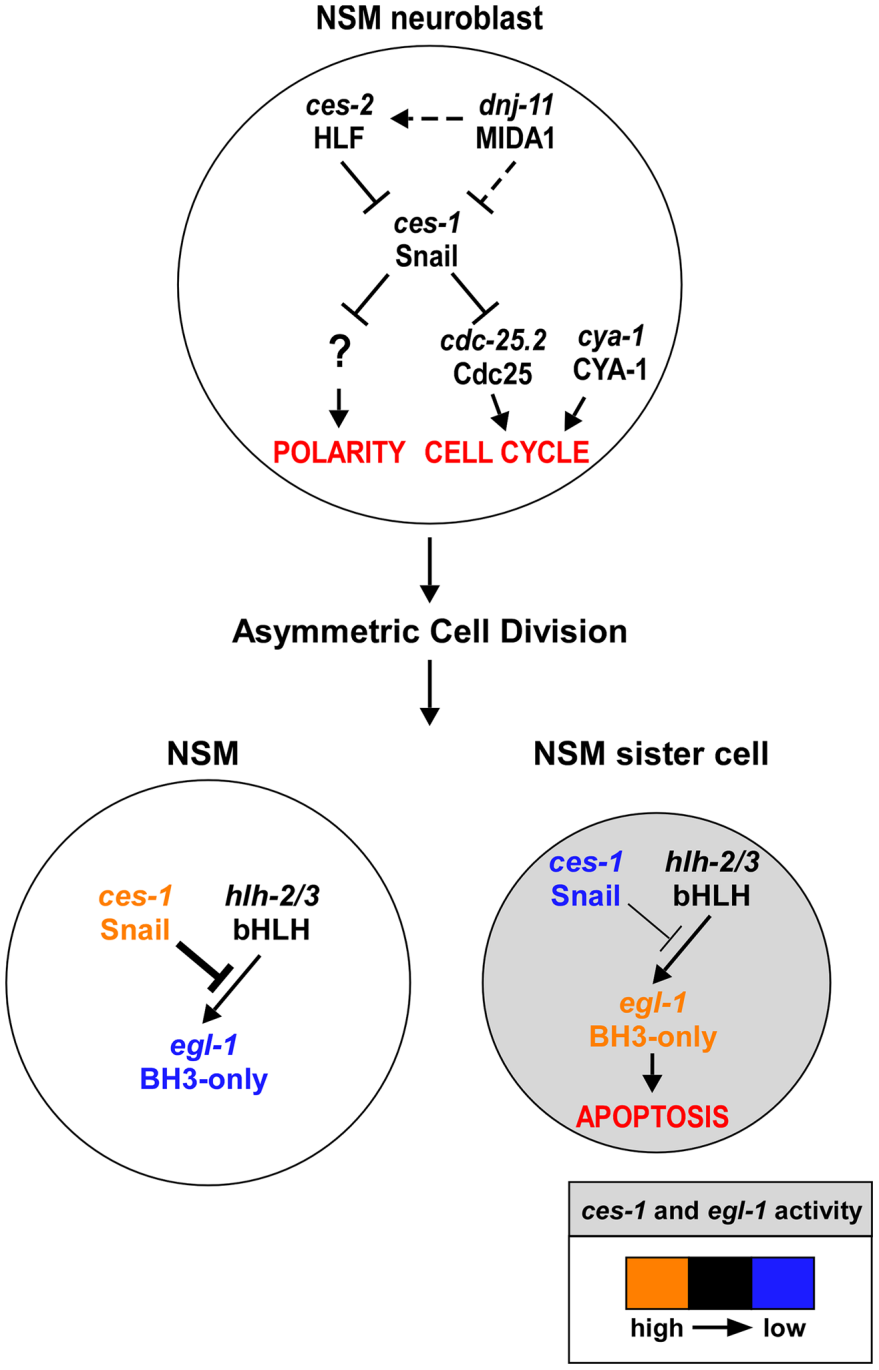
*ces-1* Snail represents a functional link between cell cycle progression, cell polarity and apoptosis in the NSM lineage. Genetic model of *ces-1* Snail functions in the NSM neuroblast (top), the NSM and the NSM sister cell (bottom). In the NSM neuroblast, *ces-1* function is negatively regulated by the genes *dnj-11* MIDA1 and *ces-2* bZIP. *ces-1* affects cell cycle progression in the NSM neuroblast by negatively regulating *cdc-25.2* Cdc25. *ces-1* also affects the polarity of the NSM neuroblast. However, to date, it is unclear through what mechanism. After the asymmetric division of the NSM neuroblast, the level of *ces-1* activity is high in the larger NSM (left) and low in the smaller NSM sister cell (right). The activity of *ces-1* in the NSM is sufficient to block the function of *hlh-2/3* bHLH, thereby resulting in a level of *egl-1* BH3-only activity that is too low to induce apoptosis. Conversely, in the NSM sister cell, the activity of *ces-1* is not sufficient to block the function of *hlh-2/3*, thereby resulting in a level of *egl-1* activity that is high enough to induce apoptosis. See text for details and molecular interpretations.

CES-1 has previously been shown to cause loss of cell polarity in the NSM neuroblast and to suppress apoptosis in its daughter cells [17], [19]. Through a combination of genetic and cell biological studies, we now show that CES-1 also affects cell cycle progression in the NSM neuroblast (See model, Figure 6). In one and the same cell lineage and within a short period of time (<150 min), CES-1 therefore impacts on at least three processes (cell cycle progression, cell polarity and apoptosis) that are fundamentally important to normal development. We speculate that it is through their ability to impact on these processes in a spatially and temporally coordinated manner that Snail-related transcription factors play a crucial role in normal development and tumorigenesis.

### The role of *C. elegans* Cyclin A in cell cycle progression

We have isolated and characterized a hypomorphic allele of the *cya-1* gene, one of two *C. elegans* cyclin A genes [31]. This *cya-1* mutation, *bc416*, presumably results in a reduction in the level of CYA-1 protein thereby causing cell division defects in specific lineages (ABarp, C, E and NSM lineages) and partially penetrant embryonic lethality. Given that animals homozygous for a *cya-1* deletion allele are not viable (S. van der Heuvel, personal communication), we conclude that *cya-1* is essential for embryogenesis. We also present evidence that *cya-1(bc416)* causes a block in cell cycle progression between S phase and M phase, which is consistent with the proposed function of the CYA-1 protein, as predicted based on the function of Cyclin A in other organisms [31], [41]. Based on the function of Cyclin A in other organisms, we also speculate that it is the *C. elegans* CDKs CDK-1 and/or CDK-2 that CYA-1 binds to and activates [31], [42]. Furthermore, we provide evidence that in the ABarp, C, E and NSM lineages, *cya-1* acts with *cdc-25.2* to cause CDK activation and, hence, cell cycle progression.

Interestingly, in *cya-1(bc416)* animals, at 15°C around 10% of the NSM neuroblast divisions are blocked, while no block in cell division was observed in the ABarp, C or E lineage (Table 1 and Figure 1C). However, at 25°C, a cell division defect was not observed for the NSM neuroblasts, while cell division defects were observed in the ABarp, C and E lineages in around 30% of the *cya-1(bc416)* embryos (Figure 1C and Figure S4). Therefore, the cell division defect in the NSM lineage is more severe at 15°C, whereas the cell division defects in the ABarp, C and E lineages are more severe at 25°C. We found that compared to wild-type animals, the reduction in the level of correctly spliced *cya-1* transcript is similar at both 15°C and 25°C in *cya-1(bc416)* animals (Figure S3E). This suggests that the splicing defect is not temperature sensitive. Why different cell lineages (NSM neuroblast, ABarp, C and E lineages) respond differently to a reduction in *cya-1* function and why their responses differ in their temperature sensitivity is unclear. Lineage or tissue-specific control of cell cycle progression has previously been observed in *C. elegans* [42]–[46]. One determining factor could be cell cycle length. Since cell cycle length is influenced by temperature and diverges greatly in different lineages, this behavior might reflect different CDK-activity thresholds for different lineages and/or differential regulation of CDKs within specific lineages at different temperatures [42]–[46].

### The role of the *dnj-11* MIDA1, *ces-2* HLF, *ces-1* Snail pathway in cell cycle progression

We propose that the *dnj-11* MIDA1, *ces-2* HLF, *ces-1* Snail pathway, which has previously been shown to control asymmetric cell division and apoptosis in the NSM lineage [17], [19], [20], also controls cell cycle progression in this lineage (Figure 6). Specifically, we demonstrate that the *dnj-11* loss-of-function mutation *tm2859* or the *ces-1* gain-of-function mutation *n703* enhances a defect in NSM neuroblast division caused by *cya-1(bc416)*. Furthermore, we have uncovered the molecular mechanism through which this pathway controls cell cycle progression in this lineage. We provide evidence in support of the notion that the Snail-related transcriptional repressor CES-1 directly represses the transcription of the *cdc-25.2* gene thereby decreasing the level of CDC-25.2. While in an otherwise wild-type background, this does not lead to a block in NSM neuroblast division, it does cause a block in NSM neuroblast division in a *cya-1(bc416)* mutant background, in which the level of CYA-1 presumably is reduced. The observation that the loss of *dnj-11* function or the *ces-1* gain-of-function mutation *n703* are synthetic lethal with *cya-1(bc416)* furthermore suggests that the *dnj-11* MIDA1, *ces-2* HLF, *ces-1* Snail pathway may act to control *cya-1*- and *cdc-25.2*-dependent cell cycle progression in lineages other than the NSM lineage.

Unlike *dnj-11(tm2859)* and *ces-1(n703*gf*)*, the *ces-1* deletion allele *tm1036* did not affect the NSM neuroblast division defect caused by *cya-1(bc416)*. This finding suggests that *ces-1* may not have a physiological role in cell cycle progression in the NSM neuroblast. Alternatively, the function of *ces-1* in cell cycle progression in the NSM neuroblast may be redundant with that of another gene or genes. Interestingly, the functions of the *D. melanogaster* Snail-related genes *snail*, *escargot* and *worniu* in cell cycle progression and polarity in embryonic neuroblasts are redundant and defects are only observed in animals in which all three genes are inactivated [9], [10]. Apart from *ces-1*, the *C. elegans* genome contains at least two additional genes that encode Snail-related transcription factors, *scrt-1* and *K02D7.2* (http://www.wormbase.org) [1]. Therefore, we speculate that the functions of *ces-1* in cell cycle progression, polarity and apoptosis in the NSM lineage are redundant with the functions in these processes of *scrt-1* and *K02D7.2*. Finally, the observation that *ces-1(n703*gf*)* enhances but *ces-1(tm1036)* partially suppresses the embryonic lethality caused by *cya-1(bc416)* supports the notion that *ces-1* has a physiological role in cell cycle progression in some cell lineages, such as the ABarp and C lineages.

### The role of Snail-related transcription factors in the regulation of cell cycle progression

Members of the Snail superfamily have previously been shown to affect cell cycle progression. Mammalian Snail1 has been shown to block cell cycle progression in cultured epithelial cells or in mouse embryos, and this effect appears to be mediated through the direct repression of *cyclin D2* transcription [15]. In contrast, over-expression of the *Snail1* gene in mouse epidermis causes hyperproliferation [47]. In *D. melanogaster*, the Snail-related proteins Snail, Escargot and Worniu have been shown to promote cell cycle progression in embryonic neuroblasts in part by, directly or indirectly, promoting *cdc25^string^* expression [10]. Cdc25*^string^* is a critical regulator of M phase during *D. melanogaster* development, whose activity is regulated at the transcriptional level [13], [14]. In support of the model that *cdc25^string^* acts as an integrator of signals that regulate cell division during *D. melanogaster* development, the *cdc25^string^* locus is subject to complex transcriptional regulation. Interestingly, it has been shown that in *D. melanogaster* larval neuroblasts, the level of Worniu has to be precisely regulated as well. A low level of Worniu in larval neuroblasts leads to a delay in cell cycle progression and premature differentiation, whereas an elevated level of Worniu results in cell cycle arrest due to increased Prospero expression [48]. These findings suggest that the roles of Snail-related proteins in cell cycle progression are complex and might be cell- or tissue-type specific.

However, so far, no Snail-related transcription factor has been implicated in the *cdc25*-mediated block of cell cycle progression. Here we have identified a new mechanism through which Snail-related proteins can block cell cycle progression. Specifically, we present evidence that, by binding to a region 4.8 kb to 6.5 kb upstream of the *cdc-25.2* transcription unit, CES-1 most likely directly represses *cdc-25.2* transcription thereby causing a block in cell cycle progression in a sensitized background (the *cya-1(bc416)* background). Interestingly, the region 4.8 kb to 6.5 kb upstream of the *cdc-25.2* transcription unit is at least partially conserved in other *Caenorhabditis* species such as *Caenorhabditis remanei* or *Caenorhabditis briggsae* (UCSC genome browser http://genome.ucsc.edu/ [49]). In analogy to *D. melanogaster cdc25^string^*, this finding suggests that the transcriptional regulation of *cdc-25.2* might be an important aspect of the developmental control of cell division in *C. elegans*, a notion that is supported by the observation that the *cdc-25.2* transcription unit is flanked by extensive intergenic regions that are conserved (UCSC genome browser [49]). As mentioned above, while *D. melanogaster* Snail, Escargot und Worniu promote cell cycle progression and *cdc25^string^* expression, *C. elegans* CES-1 blocks cell cycle progression and represses *cdc-25.2* expression. Interestingly, we found that besides repressing *cdc-25.2* transcription, the over-expression of *ces-1* directly or indirectly increases the relative level of *cdc-25.3*, which encodes another member of the Cdc25 phosphatase family of *C. elegans*. Hence, depending on the cell lineage and cellular context, members of the Snail superfamily may enhance or repress the expression of Cdc25 phosphatases.

### The role of the Snail-related transcription factors in tumorigenesis

The over-expression of Snail-related transcription factors has been implicated in the formation and progression of metastatic cancers, in part due to the ability of Snail-related transcription factors to induce EMTs [1]–[4], [50]. Their potency as proto-oncogenes is thought to lie in their capability to cause loss of cell polarity and adhesive functions on the one hand and acquisition of migratory properties on the other. We argue that their ability to, within the same cell lineage, also block cell cycle progression and apoptosis is similarly important for the formation of metastases. Cells undergoing EMT have high invasive potential and are primarily found at the margins of tumors. Whether EMT has to be accompanied by a reduction in proliferation is a question still under debate. It has previously been shown that increased expression of the gene encoding the transcription factor YB-1 (Y-box binding protein), which is frequently observed in human cancers and which results in increased *Snail1* expression, induces EMT accompanied by enhanced metastatic potential and reduced cellular proliferation [51], [52]. Here we demonstrate that the *ces-1* gf mutation not only affects cell polarity and apoptosis in the NSM lineage but also enhances the defect in cell cycle progression caused by a partial *cya-1* loss-of-function mutation. These findings support the notion that a block in cell cycle progression and, hence, cell proliferation may be important for EMT. A block in cell cycle progression could, for example, provide the time necessary for cytoskeletal reorganizations or cell polarity transitions. Finally, Snail-related transcription factors have recently been implicated in the acquisition and maintenance of the stem cell fate in mammals [4], [53]–[55]. We speculate that it is the ability of Snail-like transcription factors to coordinately influence cell cycle progression, cell polarity and apoptosis that allows specific cells to adopt and maintain the cancer stem cell fate.

Models of metastatic cancers have mainly focused on the analysis of the starting and end points of the cellular transformations that cells undergo during the formation of metastases (*i.e.* the epithelial and mesenchymal phenotype) [5]. There is a need for *in vivo* models that allow the analysis of intermediate stages of this process. We suggest that the over-expression of the Snail-related gene *ces-1* in *C. elegans* (*i.e.* the *ces-1* gf phenotype) may serve as such a model at least for certain stages of this process. The ability to combine systems biology approaches (such as ChIP-seq analyses) with cell biological and genetic dissection at single cell resolution will allow us to further dissect the complex role of Snail-like transcription factors during normal development and tumorigenesis.

## Materials and Methods

### Strains and genetics

*C. elegans* strains were maintained and cultured as described [56]. Bristol N2 was used as the wild-type strain, unless noted otherwise. CB4856 (Hawaii) was used for SNP mapping. Mutations and transgenes used in this study are listed below and are described by Riddle *et al.* unless noted otherwise [57]: LGI: *ces-1(n703*gf*), ces-1(tm1036)* (National BioResource Project) (3 times backcrossed). LGII: *rrf-3(pk1426)* [58]. LGIII: *dpy-17(e164), cya-1(bc416)* (this study) (5 times backcrossed), *bcIs66* (P*_tph-1_his-24::gfp*) (this study), *unc-69(e587), ltIs38* (P*_pie-1_gfp::ph^PLC1δ^*) [25]. LGIV: *ced-3(n717), dnj-11(tm2859)* [17]. LGV: *ltIs44* (P*_pie-1_mCherry::ph^PLC1δ^*) [25], *cdc-25.2(ok597)* [40]. LGX: *lin-15(n765*ts*)*.

### Molecular analysis

The plasmids pBC1153 (*cdc-25.2*) and pBC1282 (*cdc-25.3*) used for *in vitro* transcription of double-strand (ds) RNA were generated by cloning PCR fragments containing exons of the targeted genes into the EcoRV site of pBluescript II KS+. The plasmid pBC1098 (*cya-1*), which was used for RNAi by feeding as well as *in vitro* transcription of dsRNA, was generated by cloning a PCR fragment containing genomic DNA of the *cya-1* locus into the NcoI and XmaI sites of pPD129.36 [59]. The plasmids pMM#47 (P*_HS_ces-1*; pPD49.78 based) and pMM#48 (P*_HS_ces-1*; pPD49.83 based) were generated using a full-length *ces-1* cDNA (R.H. Horvitz and M.M. Metzstein, personal communication). For rescue experiments, DNA fragments containing the gene of interests (including regulatory regions) were amplified by PCR (NEB LongAmp Taq) and purified. The sequences of oligonucleotides used for PCR are provided in Table S5.

### RNA interference

RNAi by feeding was performed as described by Fire and co-workers [59] using 6 mM IPTG. For RNAi experiments by microinjection [60], pBC1153 (*cdc-25.2*) and pBC1282 (*cdc-25.3*) were used as templates and oligonucleotides 5′-ttgtaaaacgacggccag-3′ and 5′-catgattacgccaagcgc-3′ as primers to generate PCR products containing at their ends, either the T3 or T7 promoter. pBC1098 (*cya-1*) was used as template and oligonucleotides 5′- taatacgactcactataggg-3′ as primer to generate PCR products containing T7 promoter at both ends. These PCR products were used to synthesize dsRNA *in vitro* using T3 and T7 polymerase (Ambion). RNAi was performed by microinjection of dsRNA into young adults. Injected animals were incubated at 25°C for at least 20 h and the phenotype of their progeny was determined.

### Transgenic animals

Germline transformation was performed as described [61]. For rescue experiments, *ces-1(n703*gf*); cya-1(bc416)* animals were injected with purified PCR products (0.5–6 ng/µl) using pRF4 (100 ng/µl) as coinjection marker, which confers a dominant Rol phenotype. For *ces-1* over-expression experiment, pMM#47 (5 ng/µl) and pMM#48 (5 ng/µl) were injected into *lin-15(n765*ts*)* animals using pL15EK (80 ng/µl), which rescues the Muv phenotype caused by *lin-15(n765*ts*)*, as coinjection marker.

### Phenotypic analyses

The NSMs and the surviving NSM sister cells were identified in L3 or L4 larvae carrying the P*_tph-1_his-24::gfp* reporter using fluorescence microscopy essentially as described for P*_tph-1_gfp* [21]. The division of the NSM neuroblast was analyzed in embryos using a plasma membrane-targeted GFP fusion protein (P*_pie-1_gfp::ph^PLC1δ^*) or mCherry fusion protein (P*_pie-1_mCherry::ph^PLC1δ^*) as described [17], [25]. Embryos were imaged using 4D microscopy and cell lineage analysis was performed using a Zeiss Imager microscope and SIMIBioCell software (Simi Reality Motion Systems GmbH, Unterschleissheim, Germany) as described [29].

### DAPI staining and DNA content quantification

L4 larvae were fixed and stained with DAPI as described [62], with the exception that slides were mounted in 1.0 µg/ml DAPI in PBS diluted 1∶1 with VectaShield (Vector Laboratories). Fluorescence intensities were measured using Metamorph software. Fluorescence intensities were normalized by comparing the intensities of the nuclei of interest with the intensities of nuclei of neighboring, pharyngeal muscle nuclei. The GFP signal from the P*_tph-1_his-24::gfp* reporter was still observed after fixation and DAPI staining, and was used to identify the NSMs, NSM sister cells and non-dividing NSM neuroblasts.

### RNA preparation and cDNA synthesis

Embryos were collected and frozen at −80°C in TRIzol (Invitrogen). Frozen embryo pellets were disrupted using a 7-ml tight Dounce tissue grinder (Fisher Scientific) and total RNA was prepared using the RNeasy Mini Kit (Qiagen). The first strand cDNA synthesis reaction was performed using the SuperScript III system (Invitrogen). For cDNA synthesis, an oligo (dT) primer was used.

### *ces-1* over-expression experiment

Transgenic animals carrying an extra-chromosomal array of pMM#47, pMM#48 and the coinjection marker pL15EK were used as the sample group. Early stage embryos were isolated by bleaching synchronized hermaphrodites that contain four to six embryos. Isolated embryos were allowed to develop on large NGM plates at 20°C for 70 min. *ces-1* expression was induced by heat-shocking the embryos at 32°C for 1 h. After a 1.5 h recovery period at 20°C embryos were collected. RNA extraction and cDNA synthesis were performed as described above. Transgenic animals carrying an extra-chromosomal array of only coinjection marker pL15EK were used as control and were treated the same way.

### Quantitative real-time PCR (qPCR)

Fast SYBR green master mix (Applied Biosystems) was used to amplify cDNA templates by real-time PCR. Each sample was performed in triplicate on a Biorad CFX96 real-time PCR machine. The sequences of the primers used are provided in Table S5. The ‘housekeeping’ gene *act-1* served as endogenous control [63]. Results were analyzed using the relative standard curve method. To produce the standard curve for each target sequence including *act-1*, first, 5-fold dilution series of standard N2 cDNA were prepared and subjected to real-time PCR to determine Ct values for each dilution. Second, average Ct values were plotted versus the logarithm of the concentration (base 5) of the template. To determine the amounts of the target sequences in the starting samples, average Ct values for each sample were compared to the standard curve. To normalize the samples using *act-1*, the result of a particular target sequence was divided by the *act-1* control result.

### ChIP-seq (chromatin immunoprecipitation with massively parallel DNA sequencing)

A *ces-1::gfp* fosmid reporter was generated as described [37], [38]. Briefly, using recombineering, a GFP::3×FLAG tag was inserted in-frame to the C-terminus of the *ces-1* transcription unit in a fosmid that contains the entire locus of *ces-1*. This fosmid reporter was integrated into the worm genome using the method of bombardment, which produces transgenic animals with low-copy integrated arrays. The *in vivo* binding sites of the CES-1::GFP::3×FLAG fusion protein synthesized in these animals were then determined using the method of ChIP-seq as described [64].

## Supporting Information

Figure S1 In *ces-1(n703*gf*); cya-1(bc416)* mutants, the non-dividing NSM neuroblasts have approximately 4C DNA content. The DNA content of nuclei in *ces-1(n703*gf*); cya-1(bc416) bcIs66* animals with three GFP-positive cells was assayed by measuring the intensity of DAPI-stained nuclei. DNA content of non-dividing NSM neuroblasts, NSMs and NSM sister cells was normalized by comparing it to the DNA content of nuclei of pharyngeal muscles that have 2C DNA content. A total of 26 animals were assayed this way. Each point represents the normalized DAPI intensity of the non-dividing NSM neuroblast or the average intensity of NSM and NSM sister cell from one animal. The significance was determined by paired t-test (***p<0.001). The non-dividing NSM neuroblasts have approximately 4C DNA content (1.80), and the NSMs and NSM sister cells have 2C DNA content (0.99).
(TIF)

Figure S2 Determination of the temperature-sensitive period (TSP) of *ces-1(n703*gf*); cya-1(bc416)* and *cya-1(bc416)* animals. Embryos at different stages of embryonic development were dissected from (A) *ces-1(n703*gf*); cya-1(bc416)* and (B) *cya-1(bc416)* hermaphrodites and shifted from the permissive temperature of 15°C to the non-permissive temperature of 25°C or *vice versa*. Downshift experiments define the start of the TSP and upshift experiment the end of the TSP. About 60 embryos were assayed for each experimental set-up.
(TIF)

Figure S3 *bc416* is a mutation in the *C. elegans* Cyclin A homolog, the gene *cya-1*. (A) Genes and single-nucleotide polymorphisms (SNPs, shown in bold) used for mapping *bc416* are indicated. (B) Schematic of the *cya-1* transcription unit. Shown below is a partial sequence of the first intron (shown in small letters) and the second exon (shown in capital letters) of the *cya-1* gene. *bc416* is a G to A transition at the donor splice site (GT) in the first intron of *cya-1* as indicated by the red arrow. (C) Primer_1 was used for reverse transcriptase PCR (RT-PCR). Primer_2 that amplifies only the correctly spliced transcript of *cya-1* and Primer_3 that amplifies all transcripts of *cya-1* were used for real-time PCR (qPCR). (D) *bc416* affects the correct splicing of *cya-1*. All strains analyzed were homozygous for *bcIs66*. mRNA was extracted from mix-stage embryos of wild-type, *ces-1(n703*gf*), ces-1(n703*gf*); cya-1(bc416)* and *cya-1(bc416)* animals that were grown at 15°C and 25°C. RT-PCR products were separated using 8% polyacrylamide gels. The aberrant *cya-1* transcripts in *ces-1(n703*gf*); cya-1(bc416)* and *cya-1(bc416)* animals, are pointed out by the red arrow heads. The black arrow head points to the correctly spliced wild-type message. The aberrant bands correspond to mRNAs that retain parts of the first intron. Due to the premature stop codon in the first intron, the translation of these aberrant mRNAs would result in the synthesis of a peptide that includes only the first 12 amino acids of the full-length CYA-1 protein. (E) mRNA levels were measured by qPCR. The level of correctly spliced transcript (amplified using Primer_2) of the *cya-1* gene is normalized to the level of all the transcripts (amplified using Primer_3) of the *cya-1* gene. Data shown are the means ± SEM from at least three independent repeats. Unpaired t-test was used to determine significance. **p<0.01, ***p<0.001 significantly different.
(TIF)

Figure S4 *cya-1(bc416)* blocks cell divisions in the ABarp, C and E lineages. All strains analyzed were homozygous for *bcIs66*. Lineage analyses were performed for two (wild-type, *+/+*), three (*ces-1(n703*gf*)*) and three (*cya-1(bc416)*) embryos raised at 25°C. Cell division defects observed in three out of three embryos are depicted in red, defects found in two out of three embryos are depicted in blue, and defects found in one out of three embryos are depicted in orange. More details are provided in the legend of Figure 3.
(TIF)

Figure S5 The level of *cdc-25.3* is not changed in *cdc-25.2(RNAi)* embryos. RNAi was performed by injection of dsRNA of *cdc-25.2* into N2 young adults. Embryos were isolated from injected animals that were incubated at 25°C for 20 h. Embryos from uninjected N2 animals that were treated the same way were used as control. mRNA was extracted from embryos and mRNA levels of *cdc-25.2* and *cdc-25.3* were determined by real-time PCR (qPCR). Data are expressed as fold change relative to control. Data shown are the means ± SEM from three independent repeats. Paired t-test was used to determine significance. **p<0.01 significantly different from the control.
(TIF)

Figure S6 *cdc-25.2(RNAi)* blocks cell divisions in the ABarp, C and E lineages. Lineages analysis were performed for two (wild-type, *+/+*) and three *cdc-25.2(RNAi)* embryos raised at 25°C. For the RNAi effect, there is some variability. The lineage of *cdc-25.2(RNAi)* embryo with the strongest phenotype is shown in Figure 3, and the lineages of the other two *cdc-25.2(RNAi)* embryos are shown here (cell division defects observed in the embryo are depicted in green). More details are provided in the legend of Figure 3.
(TIF)

Table S1 *ces-1(n703*gf*); cya-1(bc416)* is not maternally rescued. All strains analyzed were homozygous for the integration *bcIs66*, and were raised and analyzed at 15°C. *ces-1(n703*gf*)* males were crossed with *ces-1(n703*gf*)*; *dpy-17(e164) cya-1(bc416)* hermaphrodites. Dpy F2 animals were scored for the number of GFP positive cells. n indicates the number of L3 or L4 larvae analyzed.
(DOC)

Table S2 *cya-1(bc416)* causes a significant reduction in brood size at 25°C. Determination of brood size at 25°C. All strains analyzed were homozygous for the integration *bcIs66*. Individual L4 larvae shifted to 25°C for 4 to 5 days. The brood size was determined by counting the eggs laid by one animal during its reproductive period. Some *ces-1(n703*gf*); cya-1(bc416), cya-1(bc416)* and *ces-1(n703*gf *n1434); cya-1(bc416)* animals were sterile after shifting to 25°C. n indicates the number of fertile animals analyzed. The brood size indicated above is the average brood size of fertile animals. ^a^ *n1434* is a *ces-1* loss-of-function allele that converts an asparagine to a stop codon. This change is predicted to produce a truncated protein lacking all five zinc-fingers [20].
(DOC)

Table S3 Comparative analysis of the 9^th^ round of division. Analysis of cell division defects by 4D lineage analysis. All strains analyzed were homozygous for *bcIs66*. Animals were raised and recordings were taken at 25°C. *cdc-25.2(RNAi)* was carried out by injection. Data shown was the analysis from two (wild-type, *+/+*) embryos, three (*ces-1(n703*gf*); cya-1(bc416)*) embryos, one (*cdc-25.2(RNAi)*) embryo that has the strongest RNAi effect (the lineage of this embryo is shown in Figure 3). The cell division in which P0 divides into AB and P1 is the 1^st^ round of division. The names presented on the left correspond to the 8 AB descendants after the 4^th^ round of division (*e.g.* ABala, ABalp). Each of them has 16 descendants after the 8^th^ round of division. In wild-type, all these 16 descendants performed the 9^th^ round of division. In *ces-1(n703*gf*); cya-1(bc416)* and *cdc-25.2(RNAi)* embryos, the number of descendants performing the 9^th^ round of division is indicated. The severe cell division defects in the ABarp lineage are observed in both *ces-1(n703*gf*); cya-1(bc416)* and *cdc-25.2(RNAi)* embryos.
(DOC)

Table S4 Comparison of the cell cycle length. Determination of cell cycle length. The Experiment was performed as described in the legend of Table S3. Data shown was the analysis from one (wild-type, *+/+*) embryo and one embryo (*cdc-25.2(RNAi)*) that has the strongest RNAi effect (the lineage of this embryo is shown in Figure 3 and Table S3). Cell cycle length is the time from the X round of division to the (X+1) round of division. ^a^ The number is the average cell cycle length and deviation in the AB lineage only. ^b^ The number is the average cell cycle length and deviation in the ABala lineage only.
(DOC)

Table S5 Primers used in this study. ^a^ The primers were used as describe [63]. ^b^ The primers were used as describe [40].
(DOC)
