# Supplementary figures and images for: Coordination of Cell Proliferation and Cell Fate Determination by CES-1 Snail
Source: PLoS Genet. 2013 Oct 31;9(10):e1003884. doi: 10.1371/journal.pgen.1003884 (PMC3814331; doi:10.1371/journal.pgen.1003884)

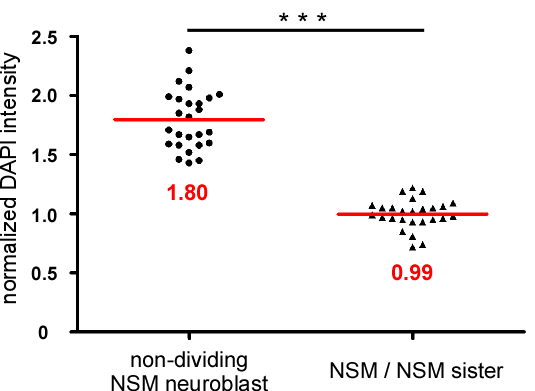

Supplement: Figure S1 — In ces-1(n703gf); cya-1(bc416) mutants, the non-dividing NSM neuroblasts have approximately 4C DNA content. The DNA content of nuclei in ces-1(n703gf); cya-1(bc416) bcIs66 animals with three GFP-positive cells was assayed by measuring the intensity of DAPI-stained nuclei. DNA content of non-dividing NSM neuroblasts, NSMs and NSM sister cells was normalized by comparing it to the DNA content of nuclei of pharyngeal muscles that have 2C DNA content. A total of 26 animals were assayed this way. Each point represents the normalized DAPI intensity of the non-dividing NSM neuroblast or the average intensity of NSM and NSM sister cell from one animal. The significance was determined by paired t-test (***p<0.001). The non-dividing NSM neuroblasts have approximately 4C DNA content (1.80), and the NSMs and NSM sister cells have 2C DNA content (0.99). (TIF) [file pgen.1003884.s001.tif]

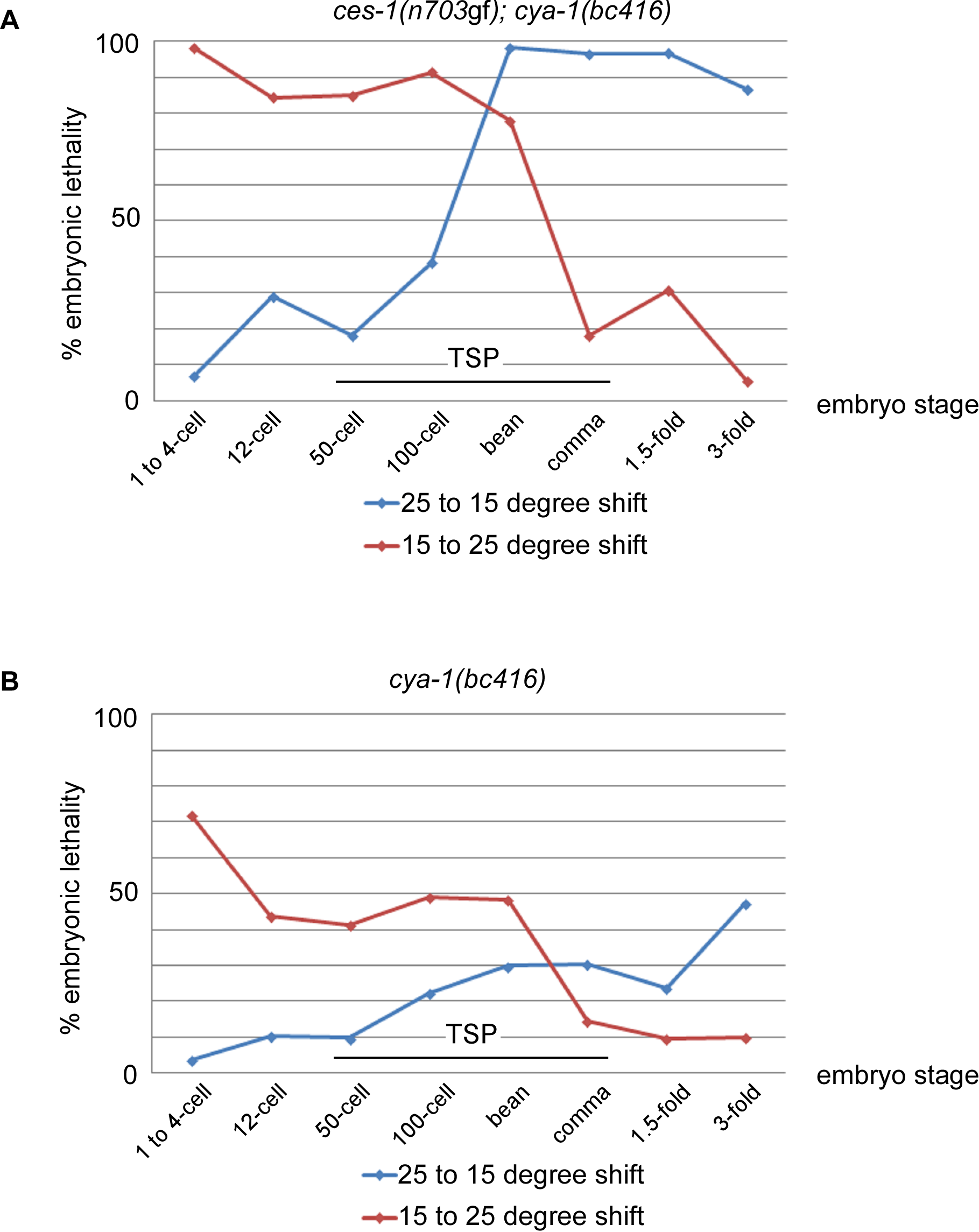

Supplement: Figure S2 — Determination of the temperature-sensitive period (TSP) of ces-1(n703gf); cya-1(bc416) and cya-1(bc416) animals. Embryos at different stages of embryonic development were dissected from (A) ces-1(n703gf); cya-1(bc416) and (B) cya-1(bc416) hermaphrodites and shifted from the permissive temperature of 15°C to the non-permissive temperature of 25°C or vice versa. Downshift experiments define the start of the TSP and upshift experiment the end of the TSP. About 60 embryos were assayed for each experimental set-up. (TIF) [file pgen.1003884.s002.tif]

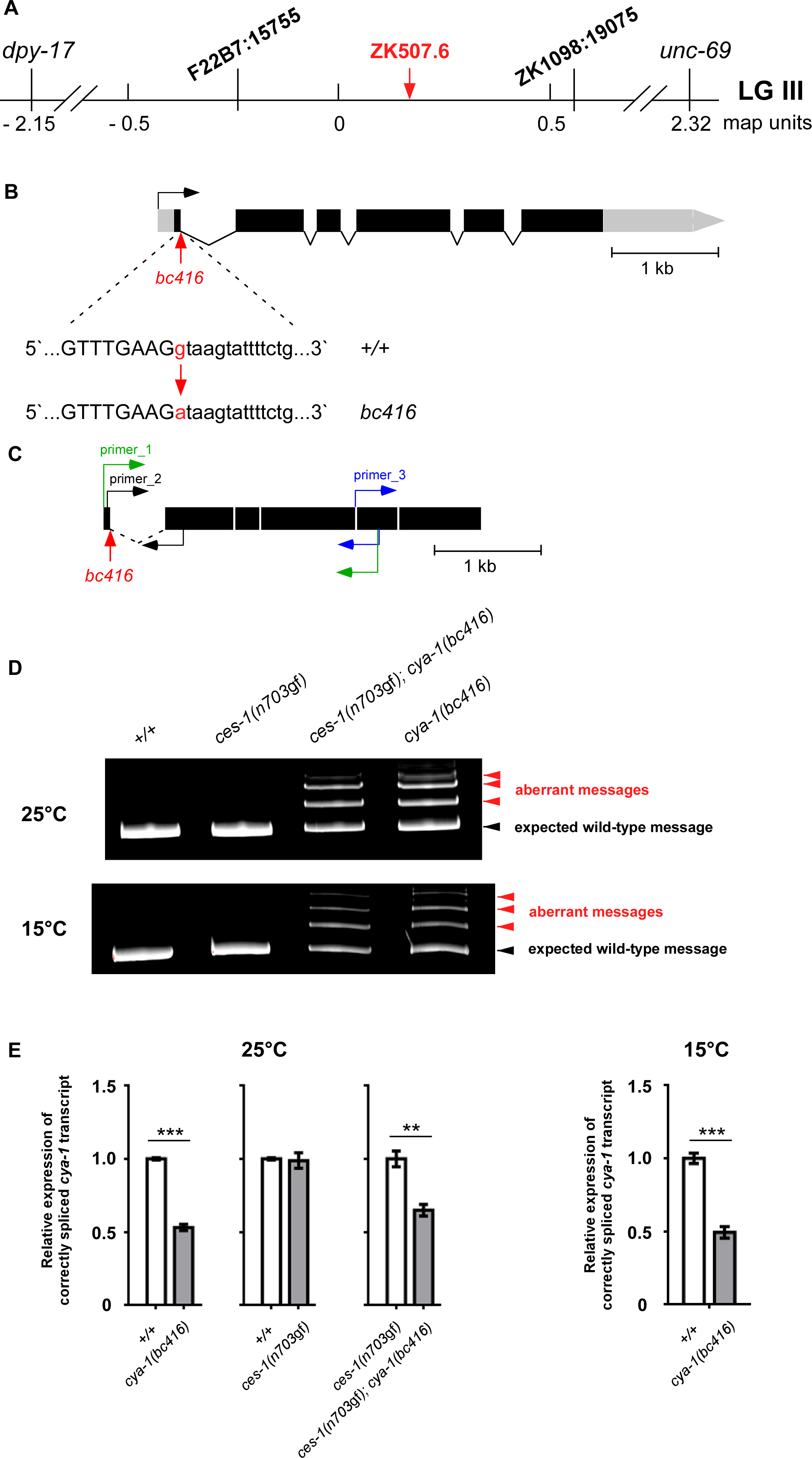

Supplement: Figure S3 — bc416 is a mutation in the C. elegans Cyclin A homolog, the gene cya-1. (A) Genes and single-nucleotide polymorphisms (SNPs, shown in bold) used for mapping bc416 are indicated. (B) Schematic of the cya-1 transcription unit. Shown below is a partial sequence of the first intron (shown in small letters) and the second exon (shown in capital letters) of the cya-1 gene. bc416 is a G to A transition at the donor splice site (GT) in the first intron of cya-1 as indicated by the red arrow. (C) Primer_1 was used for reverse transcriptase PCR (RT-PCR). Primer_2 that amplifies only the correctly spliced transcript of cya-1 and Primer_3 that amplifies all transcripts of cya-1 were used for real-time PCR (qPCR). (D) bc416 affects the correct splicing of cya-1. All strains analyzed were homozygous for bcIs66. mRNA was extracted from mix-stage embryos of wild-type, ces-1(n703gf), ces-1(n703gf); cya-1(bc416) and cya-1(bc416) animals that were grown at 15°C and 25°C. RT-PCR products were separated using 8% polyacrylamide gels. The aberrant cya-1 transcripts in ces-1(n703gf); cya-1(bc416) and cya-1(bc416) animals, are pointed out by the red arrow heads. The black arrow head points to the correctly spliced wild-type message. The aberrant bands correspond to mRNAs that retain parts of the first intron. Due to the premature stop codon in the first intron, the translation of these aberrant mRNAs would result in the synthesis of a peptide that includes only the first 12 amino acids of the full-length CYA-1 protein. (E) mRNA levels were measured by qPCR. The level of correctly spliced transcript (amplified using Primer_2) of the cya-1 gene is normalized to the level of all the transcripts (amplified using Primer_3) of the cya-1 gene. Data shown are the means ± SEM from at least three independent repeats. Unpaired t-test was used to determine significance. **p<0.01, ***p<0.001 significantly different. (TIF) [file pgen.1003884.s003.tif]

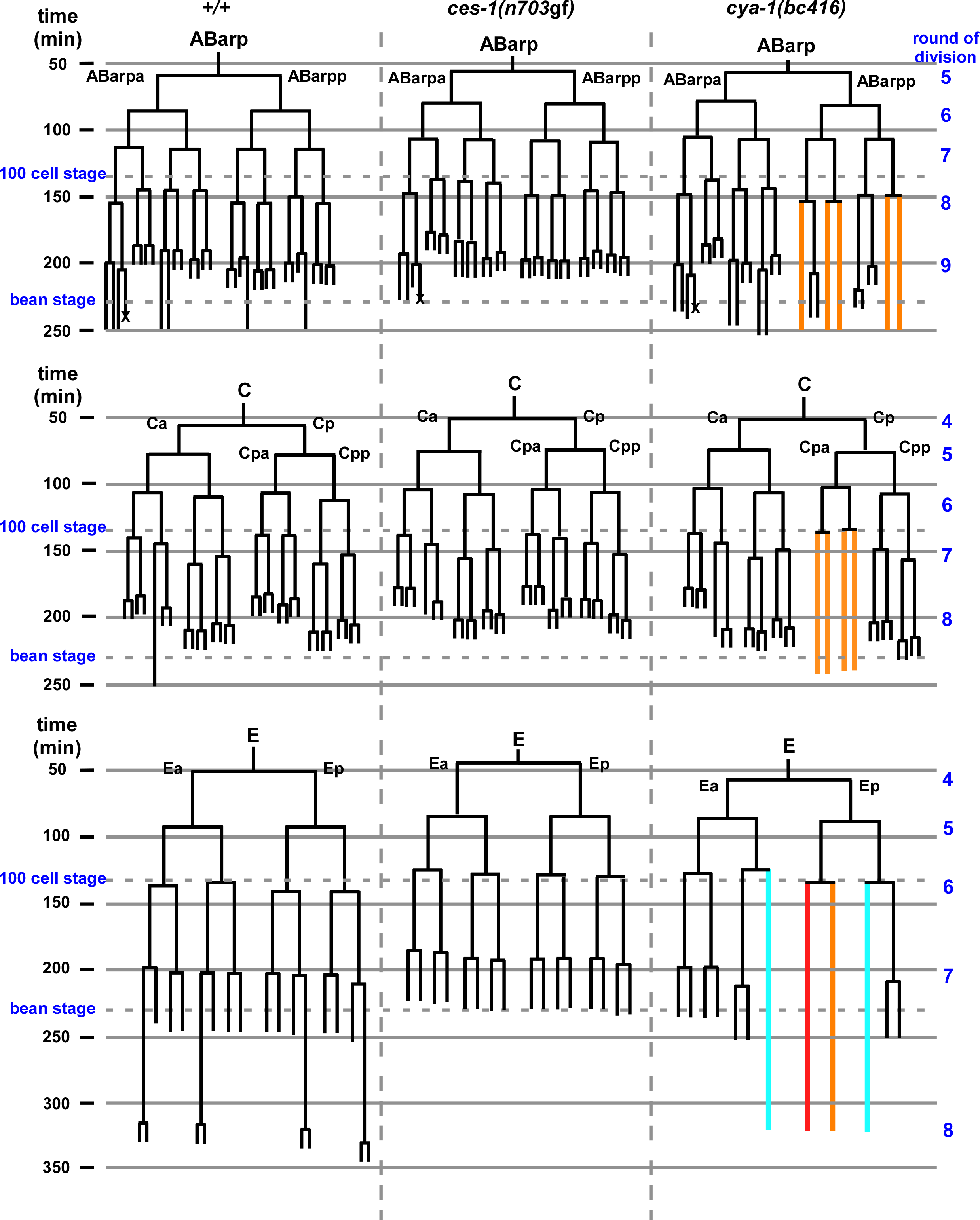

Supplement: Figure S4 — cya-1(bc416) blocks cell divisions in the ABarp, C and E lineages. All strains analyzed were homozygous for bcIs66. Lineage analyses were performed for two (wild-type, +/+), three (ces-1(n703gf)) and three (cya-1(bc416)) embryos raised at 25°C. Cell division defects observed in three out of three embryos are depicted in red, defects found in two out of three embryos are depicted in blue, and defects found in one out of three embryos are depicted in orange. More details are provided in the legend of Figure 3. (TIF) [file pgen.1003884.s004.tif]

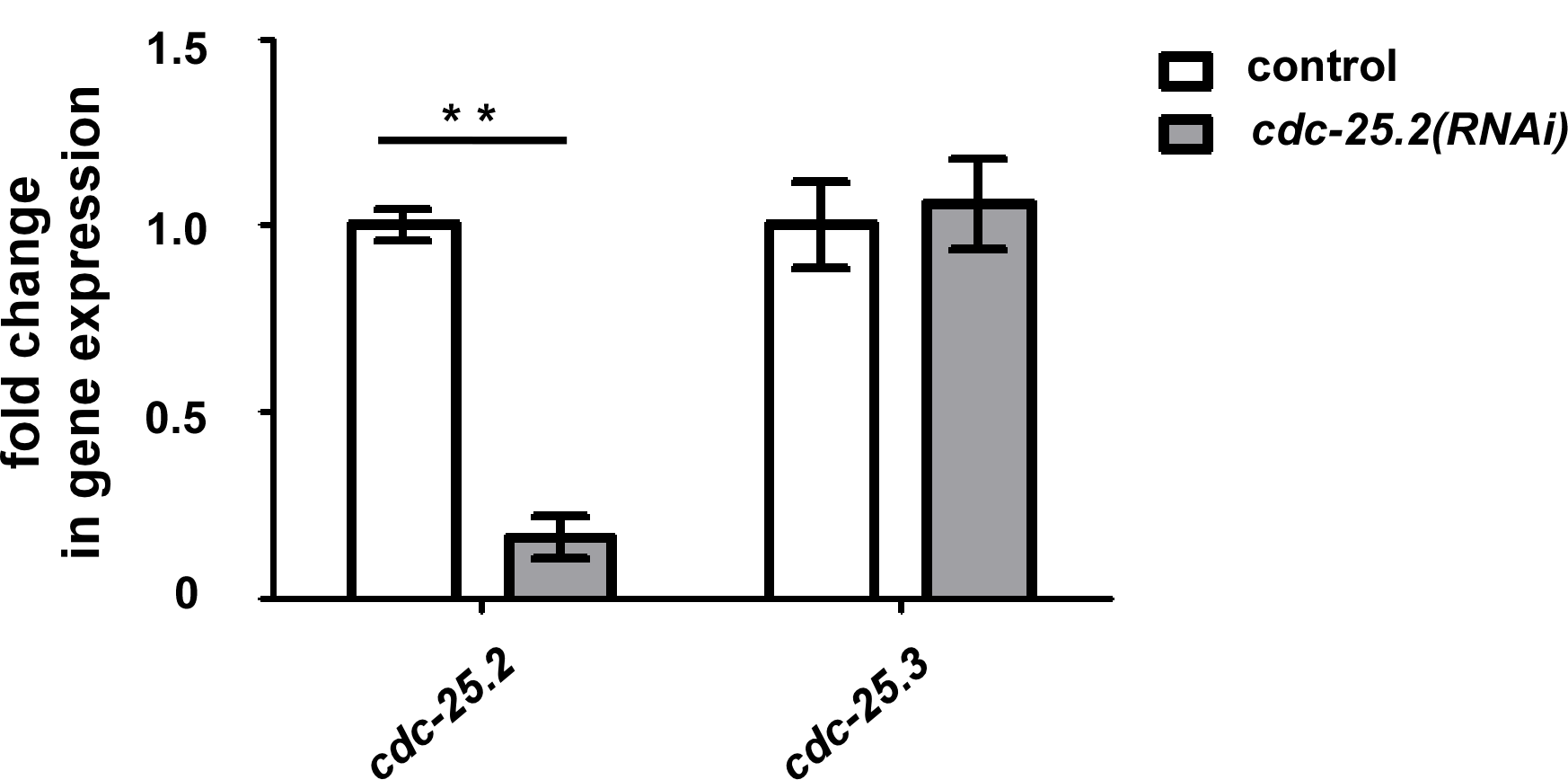

Supplement: Figure S5 — The level of cdc-25.3 is not changed in cdc-25.2(RNAi) embryos. RNAi was performed by injection of dsRNA of cdc-25.2 into N2 young adults. Embryos were isolated from injected animals that were incubated at 25°C for 20 h. Embryos from uninjected N2 animals that were treated the same way were used as control. mRNA was extracted from embryos and mRNA levels of cdc-25.2 and cdc-25.3 were determined by real-time PCR (qPCR). Data are expressed as fold change relative to control. Data shown are the means ± SEM from three independent repeats. Paired t-test was used to determine significance. **p<0.01 significantly different from the control. (TIF) [file pgen.1003884.s005.tif]

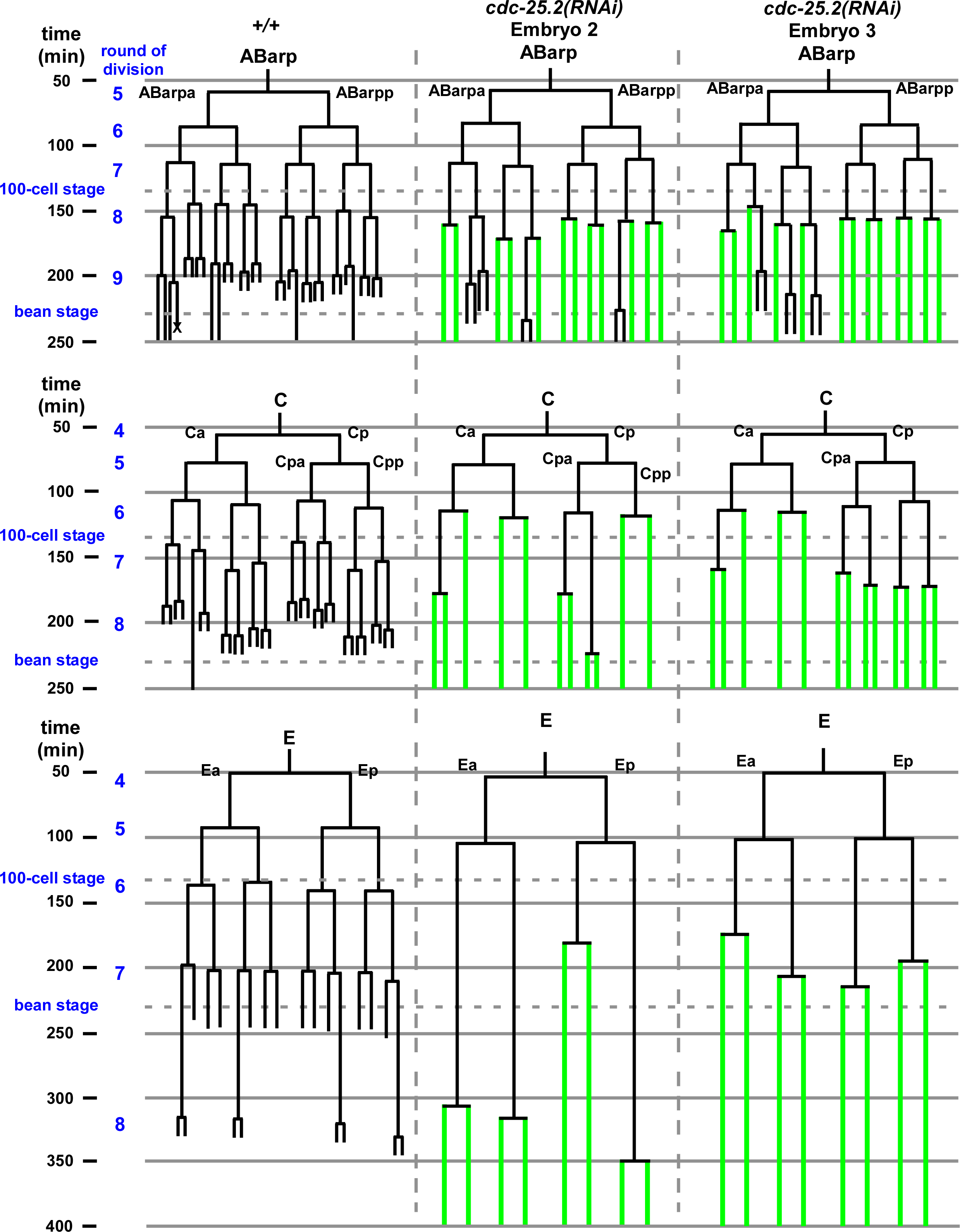

Supplement: Figure S6 — cdc-25.2(RNAi) blocks cell divisions in the ABarp, C and E lineages. Lineages analysis were performed for two (wild-type, +/+) and three cdc-25.2(RNAi) embryos raised at 25°C. For the RNAi effect, there is some variability. The lineage of cdc-25.2(RNAi) embryo with the strongest phenotype is shown in Figure 3, and the lineages of the other two cdc-25.2(RNAi) embryos are shown here (cell division defects observed in the embryo are depicted in green). More details are provided in the legend of Figure 3. (TIF) [file pgen.1003884.s006.tif]
