# Supplementary material for: Coordination of Cell Proliferation and Cell Fate Determination by CES-1 Snail
Source: PLoS Genet. 2013 Oct 31;9(10):e1003884. doi: 10.1371/journal.pgen.1003884 (PMC3814331; doi:10.1371/journal.pgen.1003884)
Supplement: Table S1 — ces-1(n703gf); cya-1(bc416) is not maternally rescued. All strains analyzed were homozygous for the integration bcIs66, and were raised and analyzed at 15°C. ces-1(n703gf) males were crossed with ces-1(n703gf); dpy-17(e164) cya-1(bc416) hermaphrodites. Dpy F2 animals were scored for the number of GFP positive cells. n indicates the number of L3 or L4 larvae analyzed. (DOC) [file pgen.1003884.s007.doc]

**Table S1. *ces-1(n703*gf*)*; *cya-1(bc416)* is not maternally rescued**

| Genotype | Maternal Genotype | n | % NSM neuroblasts dividing |
| --- | --- | --- | --- |
| *ces-1(n703*gf*); dpy-17(e164) cya-1(bc416)* | *ces-1(n703*gf*); dpy-17(e164) cya-1(bc416)* | 34 | 18% |
| *ces-1(n703*gf*); dpy-17(e164) cya-1(bc416)* | *ces-1(n703*gf*); dpy-17(e164) cya-1(bc416)/+ +* | 19 | 16% |
