# Supplementary material for: Coordination of Cell Proliferation and Cell Fate Determination by CES-1 Snail
Source: PLoS Genet. 2013 Oct 31;9(10):e1003884. doi: 10.1371/journal.pgen.1003884 (PMC3814331; doi:10.1371/journal.pgen.1003884)
Supplement: Table S2 — cya-1(bc416) causes a significant reduction in brood size at 25°C. Determination of brood size at 25°C. All strains analyzed were homozygous for the integration bcIs66. Individual L4 larvae shifted to 25°C for 4 to 5 days. The brood size was determined by counting the eggs laid by one animal during its reproductive period. Some ces-1(n703gf); cya-1(bc416), cya-1(bc416) and ces-1(n703gf n1434); cya-1(bc416) animals were sterile after shifting to 25°C. n indicates the number of fertile animals analyzed. The brood size indicated above is the average brood size of fertile animals. a n1434 is a ces-1 loss-of-function allele that converts an asparagine to a stop codon. This change is predicted to produce a truncated protein lacking all five zinc-fingers [20]. (DOC) [file pgen.1003884.s008.doc]

**Table S2. *cya-1(bc416)*** causes a significant reduction in brood size at 25°C

| Genotype | Mean Brood Size | Standard Deviation | n |
| --- | --- | --- | --- |
| *+/+* | 200 | 28 | 9 |
| *ces-1(n703*gf*)* | 182 | 58 | 8 |
| *cya-1(bc416)* | 80 | 33 | 9 |
| *ces-1(n703*gf*); cya-1(bc416)* | 91 | 56 | 15 |
| *ces-1(n703*gf *n1434); cya-1(bc416) ^a^* | 55 | 45 | 11 |
