# Supplementary material for: Coordination of Cell Proliferation and Cell Fate Determination by CES-1 Snail
Source: PLoS Genet. 2013 Oct 31;9(10):e1003884. doi: 10.1371/journal.pgen.1003884 (PMC3814331; doi:10.1371/journal.pgen.1003884)
Supplement: Table S3 — Comparative analysis of the 9th round of division. Analysis of cell division defects by 4D lineage analysis. All strains analyzed were homozygous for bcIs66. Animals were raised and recordings were taken at 25°C. cdc-25.2(RNAi) was carried out by injection. Data shown was the analysis from two (wild-type, +/+) embryos, three (ces-1(n703gf); cya-1(bc416)) embryos, one (cdc-25.2(RNAi)) embryo that has the strongest RNAi effect (the lineage of this embryo is shown in Figure 3). The cell division in which P0 divides into AB and P1 is the 1st round of division. The names presented on the left correspond to the 8 AB descendants after the 4th round of division (e.g. ABala, ABalp). Each of them has 16 descendants after the 8th round of division. In wild-type, all these 16 descendants performed the 9th round of division. In ces-1(n703gf); cya-1(bc416) and cdc-25.2(RNAi) embryos, the number of descendants performing the 9th round of division is indicated. The severe cell division defects in the ABarp lineage are observed in both ces-1(n703gf); cya-1(bc416) and cdc-25.2(RNAi) embryos. (DOC) [file pgen.1003884.s009.doc]

**Table S3. Comparative analysis of the 9^th^** round of cell division

| Cell lineage | Number of 9^th^ round of division | | |
| --- | --- | --- | --- |
|  | *+/+* | *ces-1(n703*gf*); cya-1(bc416)* | *cdc-25.2(RNAi)* |
| ABala | 16 | 16 | 10 |
| ABalp | 16 | 16 | 11 |
| ABara | 16 | 16 | 15 |
| ABarp | 16 | 10 to11 | 1 |
| ABpla | 16 | 16 | 9 |
| ABplp | 16 | 16 | 8 |
| ABpra | 16 | 16 | 12 |
| ABprp | 16 | 16 | 10 |
