# Supplementary material for: Coordination of Cell Proliferation and Cell Fate Determination by CES-1 Snail
Source: PLoS Genet. 2013 Oct 31;9(10):e1003884. doi: 10.1371/journal.pgen.1003884 (PMC3814331; doi:10.1371/journal.pgen.1003884)
Supplement: Table S4 — Comparison of the cell cycle length. Determination of cell cycle length. The Experiment was performed as described in the legend of Table S3. Data shown was the analysis from one (wild-type, +/+) embryo and one embryo (cdc-25.2(RNAi)) that has the strongest RNAi effect (the lineage of this embryo is shown in Figure 3 and Table S3). Cell cycle length is the time from the X round of division to the (X+1) round of division. a The number is the average cell cycle length and deviation in the AB lineage only. b The number is the average cell cycle length and deviation in the ABala lineage only. (DOC) [file pgen.1003884.s010.doc]

Table S4. Comparison of the cell cycle length

| Round of division | Cell cycle length | |
| --- | --- | --- |
|  | *+/+* (Mean ± SD) min | *cdc-25.2(RNAi)* (Mean ± SD) min |
| 3^rd^ to 4^th a^ | 14.5 ± 0.58 | 18.25 ± 0.5 |
| 4^th^ to 5^th a^ | 16.5 ± 1.07 | 20.63 ± 0.52 |
| 5^th^ to 6^th a^ | 24.83 ± 1.11 | 29.83 ± 1.4 |
| 6^th^ to 7^th b^ | 28.25 ± 1.5 | 36.25 ± 0.96 |
| 7^th^ to 8^th b^ | 35.88 ± 3.44 | 52.88 ± 4.05 |
