# Supplementary material for: Coordination of Cell Proliferation and Cell Fate Determination by CES-1 Snail
Source: PLoS Genet. 2013 Oct 31;9(10):e1003884. doi: 10.1371/journal.pgen.1003884 (PMC3814331; doi:10.1371/journal.pgen.1003884)
Supplement: Table S5 — Primers used in this study. a The primers were used as describe [63]. b The primers were used as describe [40]. (DOC) [file pgen.1003884.s011.doc]

**Table S5. Primers used in this study**

| Primers used in molecular cloning | | |
| --- | --- | --- |
| Plasmid | Forward sequence | Reverse sequence |
| pBC1098 | 5`-ctcccatggaaagtctccgtcgcctc-3` | 5`-tccccgggactgcgatcatcatcgac-3` |
| pBC1153 | 5`-aaggccatggttttcccccatttcgctccg-3` | 5`-aaggcccggggctcaattaccttgatgtgaccacg-3` |
| pBC1282 | 5`-ccgtcaacactcttctgcgatc-3` | 5`-gatcacatgtctgacgaagtgtcg-3` |
| Primers used in rescue experiments | | |
| gene | Forward sequence | Reverse sequence |
| *cya-1* | 5`- gcaggtataacatcttttcc-3` | 5`- agtgagagacatattactgctag-3` |
| *cdc-25.1* | 5`- gtcaacgttgacttgcgtctcg-3` | 5`- gaacccgacttgtaagttgcttgc-3` |
| *cdc-25.2* | 5`- tttggttggtaacgagagttgagg-3` | 5`- tgtcctatgacttaccacctccacc-3` |
| Primers used in RT-PCR and qPCR | | |
| gene | Forward sequence | Reverse sequence |
| *act-1^a^* | 5`-ccaggaattgctgatcgtatgcagaa-3` | 5`-tggagagggaagcgaggataga-3` |
| *cdc-25.1^a^* | 5`-atgttatcaaggtcgtctagtg-3` | 5`-ctaaatcattctccgattccgt-3` |
| *cdc-25.2^b^* | 5`-actagagacatttgaggagga-3` | 5`-gatggcgttcttgatgtgac-3` |
| *cdc-25.3* | 5`-ggcatattaagggagctcaaagcc-3` | 5`-ccacttcccgaaggttgtt-3` |
| *cdc-25.4* | 5`-tgttaagacgccagttgtgc-3` | 5`-aatggataccggcaatcaaa-3` |
| *ces-1* | 5`-ttggcaattgtgattttcaagccc-3` | 5`-cagaggtattttgtacagatgatgg-3` |
| primer_1 | 5`-gcggtcggcacttagtttgaag-3` | 5`-atgtcgggaactcggtaggt-3` |
| primer_2 | 5`-tagtttgaagccgagcaacg-3` | 5`-caattttcctcccaatggtg-3` |
| primer_3 | 5`-cgatgatgatcgcagtgaaa-3` | 5`-accaagatgatgtgggcatt-3` |
